# BioMamba: Domain-Adaptive Biomedical Language Models

**DOI:** 10.34133/hds.0454

**Published:** 2026-10-05

**Authors:** Ling Yue, Mingzhi Zhu, Sixue Xing, Yunning Cao, Yanbo Wang, Vijil Chenthamarakshan, Shaowu Pan, Payel Das, Tianfan Fu

**Affiliations:** ^1^ Department of Computer Science, Rensselaer Polytechnic Institute, Troy, NY, USA.; ^2^ Jiaxing New Jies Thermal Power Co. Ltd., Jiaxing, China.; ^3^ Department of Data Science and Technology, North University of China, Taiyuan, China.; ^4^ IBM Research, Yorktown Heights, NY, USA.; ^5^ Department of Mechanical, Aerospace, and Nuclear Engineering, Rensselaer Polytechnic Institute, Troy, NY, USA.; ^6^ State Key Laboratory for Novel Software Technology at Nanjing University, School of Computer Science, Nanjing University, Nanjing, China.

## Abstract

**Background:** Biomedical language models should improve performance on biomedical text while retaining general-language-modeling fluency. For Mamba-based models, this trade-off has not been systematically studied across biomedical literature and clinical text. **Methods:** We developed BioMamba, a family of biomedical Mamba2 models at 5 scales (130M, 370M, 780M, 1.3B, and 2.7B) obtained by continued pretraining of released public Mamba2 checkpoints on a balanced 80%/10%/10% mixture of PubMed abstracts, the Colossal Clean Crawled Corpus (C4), and Wikipedia. The architecture is unchanged from Mamba2; the contribution is the adaptation recipe and the accompanying open-weight checkpoints. We evaluated internal language modeling on fixed held-out sets, out-of-domain multiple-choice benchmarks, and 3 downstream tasks across multiple model scales: clinical note completion and discharge summary generation on MIMIC-IV-Note, and biomedical yes/no question answering on Biomedical Semantic Indexing and Question Answering (BioASQ) and PubMed Question Answering (PubMedQA). **Results:** Across 5 scales, BioMamba consistently lowered PubMed perplexity, improved Wikipedia-style held-out perplexity by 1.46 to 4.72 PPL, and left C4 perplexity essentially unchanged (∣ΔPPL∣≤0.17). On 6 out-of-domain multiple-choice benchmarks, BioMamba stayed within ±3 pp of Mamba2 with no systematic regression. After supervised fine-tuning, BioMamba+SFT matched or exceeded Mamba2+SFT on MIMIC-IV note completion and discharge summary generation at every evaluated scale (paired-bootstrap $P\left(\Delta >0\right)\geq 0.997$ at 2.7B) and improved PubMedQA at every scale. The strongest model (BioMamba-2.7B) reached a PubMed perplexity of 5.28 and accuracies of 90.24% and 73.00% on BioASQ and PubMedQA, respectively. **Conclusions:** A balanced domain-adaptive continued pretraining recipe strengthens Mamba2 language models on biomedical literature and clinical text while preserving general-language-modeling fluency.

## Introduction

Biomedical research and health data science increasingly rely on unstructured text, including PubMed abstracts, full-text articles, clinical guidelines, trial reports, and clinical documentation. Domain-adapted language models are therefore important for biomedical information extraction, evidence synthesis, summarization, completion, and question answering [1]. Transformer-based biomedical models such as BioBERT [2], SciBERT [3], PubMedBERT [4], BioGPT [5], SciFive [6], and BioBART [7] have shown that adapting general-purpose language models to biomedical corpora can yield strong performance. In clinical natural language processing, ClinicalBERT [8], GatorTron [9], and ClinicalT5 [10] further demonstrate the value of domain adaptation for clinical text. Recent work in health data science and public-health artificial intelligence has also emphasized that models used in biomedical workflows should be accurate, transparent, and practically useful [11–16].

Most biomedical language models still use Transformer self-attention, which has quadratic cost in sequence length [17]. This cost becomes restrictive when biomedical inputs are long, terminology-dense, and distributed across multiple sentences or sections. State space models, especially structured state space models [18], offer an alternative. Mamba introduced selective state space layers for linear-time sequence modeling, and later work further strengthened the connection between state space models and attention while preserving favorable scaling for long contexts [19,20].

Despite this progress, biomedical adaptation of Mamba models remains limited. Biomedical language modeling is still dominated by Transformer families and related open medical language models [2,4,5,21–24], and the few reported Mamba-based biomedical adaptations have focused on narrower settings such as clinical notes [25]. Second, domain adaptation risks catastrophic forgetting: A model may improve on biomedical text while losing general-language-modeling fluency, which is a particular concern since biomedical writing often mixes specialized terminology with broader scientific language. This trade-off has been documented in domain-adaptive pretraining literature [26,27] and in work explicitly targeting general knowledge preservation [28].

We address this gap with BioMamba, a family of biomedical models built by continued pretraining of released Mamba2 checkpoints. Our contribution is a reproducible biomedical continued pretraining recipe together with a family of open-weight BioMamba checkpoints at 130M, 370M, 780M, 1.3B, and 2.7B parameters; the architecture itself is unchanged from Mamba2. The main focus is therefore the adaptation strategy, its empirical effects, and the accompanying resource release rather than an architectural modification. This study makes 3 contributions:

• A public release of the training and evaluation code and all 5 checkpoints at https://github.com/LeoYML/BioMamba under the Apache-2.0 license, so that the biomedical natural language processing community can directly use or extend the models.
• A systematic investigation of the continued pretraining pipeline showing that an appropriate 80%/10%/10% PubMed/Colossal Clean Crawled Corpus (C4)/Wikipedia mixture can enhance biomedical language modeling while preserving general-language-modeling fluency and mitigating catastrophic forgetting.
• Controlled base-versus-adapted experiments showing that BioMamba achieves consistent improvements across downstream tasks, including biomedical question answering (PubMed Question Answering [PubMedQA] and Biomedical Semantic Indexing and Question Answering [BioASQ]), clinical note completion, and discharge summary generation on MIMIC-IV.

## Methods

### Overview

This study tests whether biomedical continued pretraining [26,27] can improve Mamba language models while retaining general-language-modeling fluency. Figure 1 shows the overall workflow. We started from public Mamba2 checkpoints, continued pretraining on a mixed corpus of PubMed, C4, and Wikipedia, evaluated language-model quality on fixed in-domain and out-of-domain validation sets, and then fine-tuned the resulting models on clinical note completion, discharge summary generation, and biomedical question answering.

**Fig. 1.**
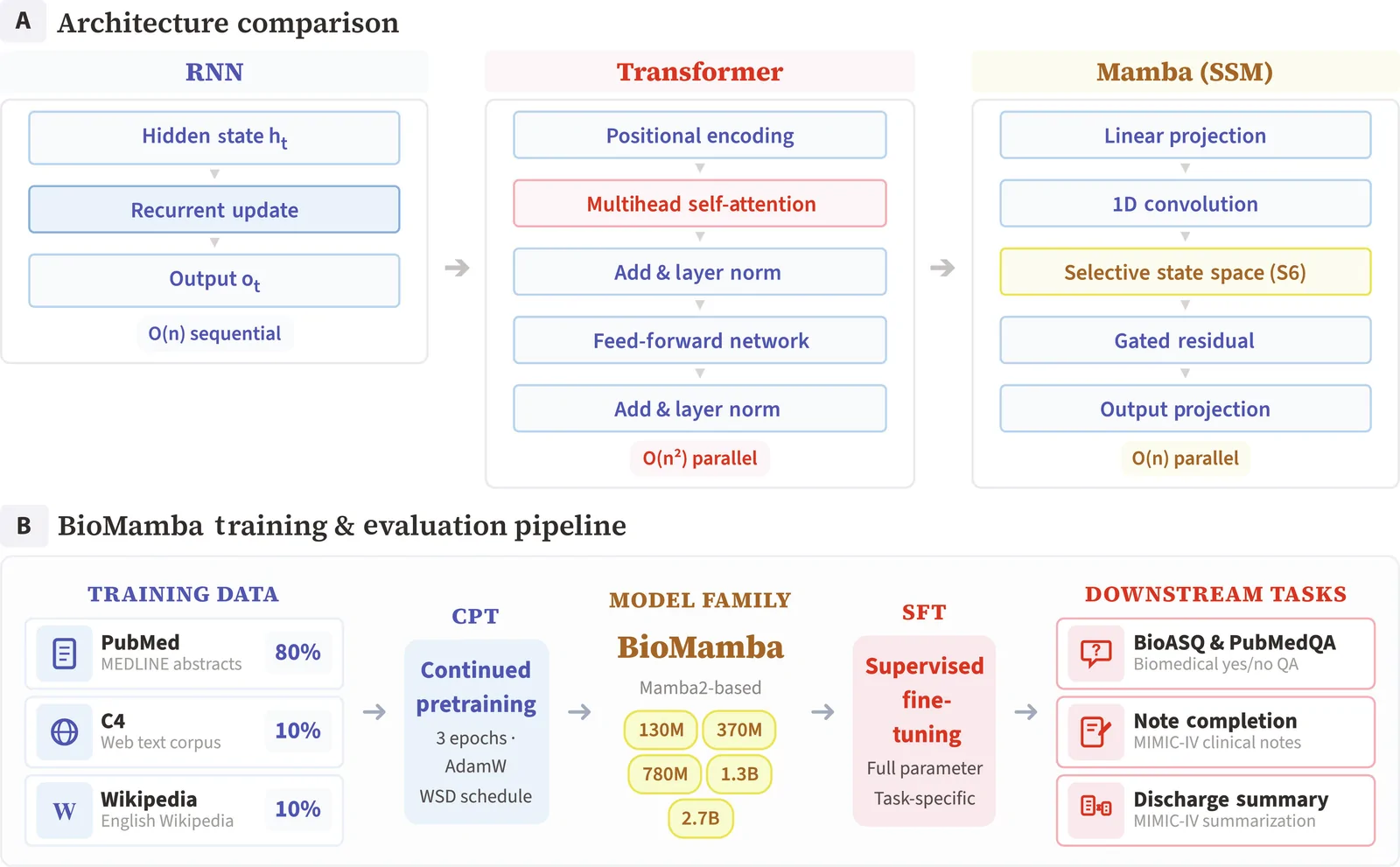
Overview of BioMamba. (A) Architecture comparison: recurrent neural network (RNN), Transformer, and Mamba (state space model [SSM]), with key distinguishing components highlighted. (B) Training and evaluation pipeline: Continued pretraining on a mixed PubMed/Colossal Clean Crawled Corpus (C4)/Wikipedia corpus produces BioMamba models at 5 scales (130M to 2.7B), which are then fine-tuned for biomedical question answering, clinical note completion, and discharge summary generation. WSD, Warmup–Stable–Decay learning-rate schedule.

Before analysis, we specified which comparisons would be treated as primary. The primary comparison is between each base model and its BioMamba counterpart under a shared tokenizer and fixed evaluation sets. These comparisons isolate the effect of biomedical adaptation most directly. Comparisons with public checkpoints are included only for context because tokenizers and prior training histories differ across released models.

### Model family and training data

BioMamba was obtained by continued pretraining of released Hugging Face Mamba2 checkpoints from state-spaces/mamba2-* [19,20] at 5 model scales spanning 130M to 2.7B parameters. To keep tokenization fixed across internal comparisons, all models use the shared GPT-NeoX tokenizer [29] from state-spaces/mamba-2.8b-hf, which has a vocabulary of 50,280 tokens.

All BioMamba variants were trained autoregressively with the standard next-token objective,

$$L_{\mathrm{LM}}=-\sum_{t=1}^{T}\log P\left(x_{t}|\;x_{<t};\;\theta \right),$$ (1)

where $x_{t}$ is the token at position *t*, $x_{<t}$ denotes the preceding context, and θ represents model parameters.

PubMed abstracts indexed in Medical Literature Analysis and Retrieval System Online (MEDLINE) provided the biomedical data [30], and we added C4 [31] and English Wikipedia [32] in smaller proportions as the general-domain component of the mixture. After tokenization, filtering, and shuffling, the final corpus contained approximately 508K sequences of length 1,024 tokens (Table 1). Mixture selection was performed in a separate 130M pilot ablation before retraining the final 80%/10%/10% configuration used for the main BioMamba checkpoints.

**Table 1.** Continued pretraining data composition. C4 denotes the Colossal Clean Crawled Corpus.

Component	Source	Proportion	Sequences
PubMed	Abstracts indexed in MEDLINE (1,024-token sequences)	~80%	~424K
C4	General-domain web text	~10%	~42K
Wikipedia	English Wikipedia	~10%	~42K
**Total**		**100%**	**~508K**

MEDLINE, Medical Literature Analysis and Retrieval System Online

### Training procedure

Continued pretraining was performed for 3 epochs on the mixed PubMed/C4/Wikipedia corpus using AdamW [33] optimization with weight decay 0.1, BF16 mixed precision, a maximum sequence length of 1,024 tokens, gradient clipping of 1.0, and random seed 42. To keep optimization roughly comparable across scales, we adjusted per-device microbatch size and gradient accumulation so that the effective batch size stayed approximately constant, at 240 to 256 sequences for the 130M–1.3B models and 192 sequences for the 2.7B model. This yielded 5,967 to 7,953 optimizer steps over the full 3-epoch training budget. Exponential moving average of model weights was enabled throughout training, and the checkpoint with the lowest validation loss was kept for downstream evaluation.

To mitigate catastrophic forgetting, we employed a conservative warmup–stable–decay schedule together with layer-wise learning-rate decay, which updates lower layers more conservatively than higher layers using a layer-wise decay factor of 0.90 at 130M and 0.95 for the larger models. Peak learning rates decreased with model size, from 1.5 × 10^−6^ at 130M to 2.0 × 10^−7^ at 2.7B (4.5 × 10^−7^ for 370M, 3.0 × 10^−7^ for 780M, and 2.5 × 10^−7^ for 1.3B).

Shared and model-specific implementation details, including exact scheduler configuration, per-scale microbatch settings, hardware allocation, and memory usage, are reported in Section S2. As shown later in Results, training remained stable across all scales, with smooth decreases in validation loss and the best validation point occurring at or near the final step in every run.

### Evaluation setup

#### Internal language-model evaluation

The primary language-model evaluation used fixed validation sets of 1,000 held-out sequences for PubMed [30] and C4 [31], drawn from the same data pools as continued pretraining but excluded from the training split, and tokenized with the shared tokenizer. For the Wikipedia held-out set, we used the Wikitext-103 raw split, referred to throughout this paper as the “Wikipedia-style held-out” set for brevity, and tokenized it with the same shared tokenizer. This is the most controlled comparison in the study because the tokenizer is held constant across all internal evaluations and each benchmark split is fixed in advance.

#### Contextual comparison with public checkpoints

To place BioMamba alongside released biomedical and medically adapted checkpoints, we also evaluated public models on the same raw text using each model’s native tokenizer. Because perplexity depends on tokenization, these results are contextual rather than strictly comparable and are interpreted only as secondary model-level evidence. Exact checkpoint sources are listed in Section S3.

### Downstream tasks

We evaluated downstream transfer to clinical text on 2 complementary supervised generation tasks constructed from deidentified discharge summaries in MIMIC-IV-Note v2.2 [34] within the broader MIMIC-IV resource [35]. Note completion evaluates continuation ability: The model receives the first half of a discharge note and generates its continuation. Discharge summary generation evaluates summarization ability: The model receives structured admission sections and generates discharge sections. To avoid information leakage, splits were defined at the patient level, and quantitative evaluation used 500 randomly sampled held-out test notes per task. These clinical generation experiments were conducted at all 5 model scales. The 2 clinical generation settings are closely related to prior work on hospital-course summarization and discharge-note generation [36–40]. Full data construction, fine-tuning settings, and qualitative examples are provided in Sections S5 and S6.

We also evaluated downstream transfer on biomedical yes/no question answering [1] because it provides a simple and interpretable test of whether the model can use biomedical knowledge after continued pretraining. Supervised fine-tuning (SFT) used a mixture of BioASQ yes/no training examples from the BioASQ challenge series [41–43] aggregated from BioASQ 7B [44] and 13B [45] (1,114 samples after class balancing) and a binary yes/no split derived from the PubMedQA dataset [46] (800 training samples). Held-out evaluation was conducted on BioASQ 13B and the corresponding 200-sample binary PubMedQA test split. For both downstream settings, SFT updated all model parameters rather than using parameter-efficient adapters.

### Metrics and analysis

For language modeling, we report cross-entropy-derived perplexity (PPL),

$$\mathrm{PPL}=\exp\left(\bar{L}\right),$$ (2)

where $\bar{L}$ is the mean next-token cross-entropy on the evaluation set. Lower PPL indicates better language modeling. For downstream question answering, we report accuracy and macro-F1 score, where the macro-F1 score is the arithmetic mean of the class-specific yes and no F1 scores. Class-specific yes and no F1 scores are reported when helpful to show which class benefited most from continued pretraining.

For clinical text generation, note completion is treated as a completion task and discharge summary generation as a summarization task. For both, we report Recall-Oriented Understudy for Gisting Evaluation (ROUGE): ROUGE-1, ROUGE-2, and ROUGE-L [47]. ROUGE-*n* measures the *n*-gram recall between the generated text and the reference:

$$\begin{array}{c} \text{ROUGE}-n=\frac{\sum_{s\in R}\sum_{{\text{gram}}_{n}\in s}{\text{Count}}_{\text{match}}\left({\text{gram}}_{n}\right)}{\sum_{s\in R}\sum_{{\text{gram}}_{n}\in s}\text{Count}\left({\text{gram}}_{n}\right)}, \end{array}$$ (3)

where R is the set of reference sentences and ${\text{Count}}_{\text{match}}$ counts the number of *n*-grams that appear in both the generated and reference texts. ROUGE-L uses the longest common subsequence (LCS) instead of fixed-length *n*-grams:

$$\text{ROUGE}-L=\frac{\left(1+\beta^{2}\right)\cdot P_{\mathrm{lcs}}\cdot R_{\mathrm{lcs}}}{R_{\mathrm{lcs}}+\beta^{2}\cdot P_{\mathrm{lcs}}},$$ (4)

where $P_{\mathrm{lcs}}=\mathrm{LCS}\left(X,\;Y\right)/|\;X\;|$ and $R_{\mathrm{lcs}}=\mathrm{LCS}\left(X,\;Y\right)/|Y|$ are LCS-based precision and recall and β is set to favor recall. Higher ROUGE scores indicate greater overlap with the reference text.

For downstream accuracy, we report 95% Wilson binomial confidence intervals in the text when helpful to reflect the small size of the BioASQ test set (*n* = 82).

## Results

### Biomedical language modeling

#### Quantitative results

Under the tokenizer-controlled internal comparison, continued pretraining lowered PubMed perplexity at every model scale (Table 2 and Fig. 2).

**Table 2.** Internal perplexity comparison between base Mamba2 models and BioMamba. The base models are the public checkpoints used for initialization. Boldface indicates a perplexity lower (better) than the corresponding base Mamba2 checkpoint.

Model	PubMed PPL (↓)	Wikipedia PPL (↓)	C4 PPL (↓)
Mamba2-130M (base)	9.41	23.09	26.58
BioMamba-130M	**8.42**	**18.76**	26.58
Mamba2-370M (base)	7.41	16.17	20.06
BioMamba-370M	**6.75**	**13.36**	**20.01**
Mamba2-780M (base)	6.61	13.54	17.32
BioMamba-780M	**6.15**	**11.58**	17.32
Mamba2-1.3B (base)	6.12	11.91	15.59
BioMamba-1.3B	**5.66**	**10.04**	**15.43**
Mamba2-2.7B (base)	5.67	10.28	13.99
BioMamba-2.7B	**5.28**	**8.85**	**13.87**

C4, Colossal Clean Crawled Corpus; PPL, perplexity

**Fig. 2.**
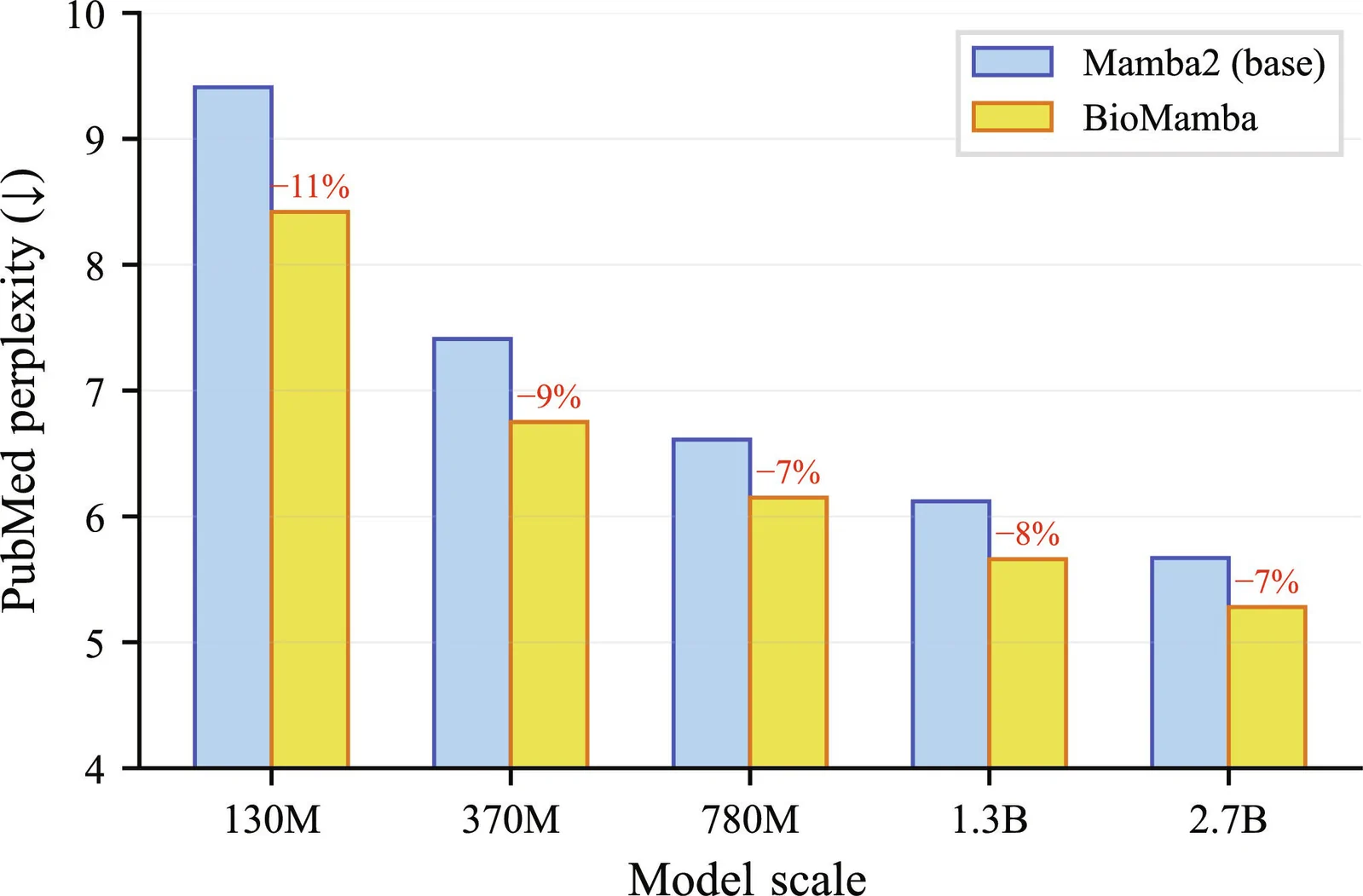
PubMed perplexity comparison between base Mamba2 models and BioMamba across 5 model scales. Percentage labels indicate the relative perplexity reduction achieved by continued pretraining at each fixed model scale. The within-scale gap between each base model and its BioMamba counterpart is largest at 130M, indicating that the marginal benefit of continued pretraining is most pronounced for the smallest model.

Continued pretraining yielded consistent reductions in PubMed perplexity across all scales, dropping from 9.41 to 8.42 in the 130M model and scaling down to 5.28 in the 2.7B model.

The relative PubMed perplexity reduction ranged from 7% to 11% across scales, demonstrating that biomedical adaptation yields consistent gains even at the largest model size.

Wikipedia-style held-out perplexity also decreased at every scale, consistent with the inclusion of Wikipedia in the continued pretraining mixture. By contrast, C4 perplexity changed by at most about 1%, so PubMed gains did not come at the cost of out-of-domain C4 performance before any task-specific fine-tuning.

#### Case study

The smallest model, BioMamba-130M, shows the main pattern clearly. Continued pretraining lowered PubMed perplexity from 9.41 to 8.42 and Wikipedia-style held-out perplexity from 23.09 to 18.76, while C4 perplexity remained 26.58. This example shows that the mixed-corpus strategy improved biomedical language modeling without harming the general-language-modeling fluency measured on C4.

#### Contextual comparison with public checkpoints

Table 3 reports raw-text perplexity for BioMamba alongside released biomedical and medically adapted checkpoints, each evaluated with its own native tokenizer. Because tokenizers, pretraining data, and adaptation protocols differ, we use these results only to situate BioMamba within the released-model landscape rather than as a controlled architecture-level comparison; within that contextual view, BioMamba-2.7B falls among the strongest raw-text perplexities in this set, while BioMamba-130M remains in a competitive range at a much smaller scale.

**Table 3.** Raw-text perplexity comparison with released biomedical and medically adapted language models, each evaluated on the same text with its own native tokenizer

Model	# Parameters	PubMed PPL (↓)	Wikipedia PPL (↓)	C4 PPL (↓)
BioMamba-130M (ours)	130M	11.08	20.49	29.01
BioGPT	347M	15.09	61.31	104.62
Bio-Medical-Llama-3.2-1B	1B	14.46	22.54	36.42
Gemma3-1B (fine-tuned)	~1B	19.90	35.01	41.67
BioGPT-Large	1.5B	10.00	35.83	50.79
BioGPT-Large-PubMedQA	1.5B	9.26	43.54	71.96
Meditron3-Gemma2-2B	2B	6.67	11.61	15.62
BioMedLM	2.7B	25.27	100.24	95.74
BioMamba-2.7B (ours)	2.7B	6.52	9.71	14.91

C4, Colossal Clean Crawled Corpus; PubMedQA, PubMed Question Answering; PPL, perplexity

### Out-of-domain evaluation and evaluation-budget stability

To evaluate whether the 80%/10%/10% recipe preserves general-language-modeling fluency beyond the in-domain perplexity benchmarks, we added 2 complementary analyses. First, we evaluated all 5 BioMamba checkpoints and their Mamba2 base counterparts on 6 out-of-domain multiple-choice benchmarks (LAMBADA, HellaSwag, ARC-Easy, ARC-Challenge, PIQA, and OpenBookQA), scored by continuation log-likelihood. Across tasks and scales, BioMamba stays within ±3 pp of Mamba2 on every task with no systematic regression (Table 4); the per-scale mean change ranges only from −0.51 to +0.58 pp, which is consistent with the absence of catastrophic forgetting on broad commonsense reasoning, reading comprehension, and elementary-science benchmarks.

**Table 4.** Out-of-domain multiple-choice summary across 6 benchmarks. For each scale, we report the mean BioMamba-Mamba2 accuracy difference averaged over the 6 tasks and the range. All per-task Δ lie inside ±3 pp at every scale. The full accuracy grid is reported in Table S14.

Scale	Mean Δ (pp)	Per-task Δ range (pp)
130M	−0.22	[−2.10, +0.80]
370M	+0.22	[−1.60, +1.67]
780M	−0.51	[−1.20, +0.53]
1.3B	+0.58	[−0.33, +2.36]
2.7B	+0.10	[−0.67, +0.60]

Second, we checked that the perplexity numbers in the “Biomedical language modeling” section remain stable when the evaluation budget is increased from 1,024 to 2,048 and 4,096 tokens. We therefore re-evaluated C4, Wikitext-103, and PubMed-medline at evaluation budgets of 1,024, 2,048, and 4,096 tokens. Across all scales and budgets, values agree within ±0.01 PPL; Table 5 therefore reports a representative single column rather than a 3-column grid (the 2,048 column is representative of 1,024 and 4,096 under this rounding). The Mamba2→BioMamba deltas are ∣ΔPPL∣≤0.17 on C4, −1.46 to −4.72 PPL on Wikitext-103, and −0.11 to −0.44 PPL on PubMed-medline, with the Wikitext-103 gain largest at the smaller scales and decreasing monotonically with model size—consistent with the finding in the “Biomedical language modeling” section that continued pretraining is especially helpful in the lower-cost regime.

**Table 5.** Extended-budget perplexity for Mamba2 base and BioMamba on C4, Wikitext-103, and PubMed-medline. Each numeric cell shows Mamba2→BioMamba with the delta in parentheses. Boldface indicates the lowest (best) perplexity in each row.

Scale	C4 PPL	Wikitext-103 PPL	PubMed PPL
130M	22.42→22.40 (−0.02)	23.42→**18.70** (−4.72)	11.67→11.23 (−0.44)
370M	17.09→17.05 (−0.04)	16.41→**13.61** (−2.80)	9.06→8.80 (−0.26)
780M	14.84→14.81 (−0.03)	13.66→**11.62** (−2.04)	7.99→7.85 (−0.14)
1.3B	13.37→13.25 (−0.12)	12.03→**10.06** (−1.97)	7.40→7.26 (−0.14)
2.7B	12.10→11.97 (−0.13)	10.36→**8.90** (−1.46)	6.80→6.69 (−0.11)

C4, Colossal Clean Crawled Corpus; PPL, perplexity

### Clinical note completion and discharge summary generation on MIMIC-IV

We next asked whether biomedical adaptation learned from PubMed would transfer to clinical documentation. We therefore evaluated the models on MIMIC-IV-Note v2.2, using the patient-level split and 500-sample held-out evaluation described in Methods and in Section S5.

#### Clinical SFT

Starting from both the Mamba2 base checkpoints and the BioMamba checkpoints, we performed clinical SFT on 2 generation tasks constructed from the discharge notes. Training data comprised up to 20,000 note-completion samples and approximately 12,893 discharge-generation samples (32,893 total; 90% train, 10% validation). Instruction masking was applied so that only response tokens contributed to the loss. BioMamba+SFT uses a lower learning rate and more training epochs than Mamba2+SFT because aggressive fine-tuning tends to overwrite the information acquired during continued pretraining; to verify that this design choice does not create an unfair comparison, we additionally ran a matched-budget control in which Mamba2 was fine-tuned with the exact BioMamba SFT recipe, and the BioMamba schedule did not close the gap (Section S9). We also found that validation loss was not a reliable proxy for downstream ROUGE in the BioMamba setting, which further motivates the conservative BioMamba SFT schedule. Full SFT data construction, hyperparameter configurations, and tuning details are provided in Section S5.

#### Evaluation tasks

We evaluated 2 clinical generation tasks that reflect different capabilities. In note completion, the model receives the first half of a discharge note and generates a 128-token continuation (greedy decoding), scored against the ground-truth second half by ROUGE-1, ROUGE-2, and ROUGE-L. In discharge summary generation, the model receives structured admission sections and generates discharge sections, again scored by ROUGE.

#### Results

Table 6 presents MIMIC-IV ROUGE across all 5 scales, with the Mamba2+SFT / BioMamba+SFT label convention used throughout. For the 2.7B row, we additionally report, in Section S14, (a) per-arm 95% percentile-bootstrap confidence intervals for ROUGE-1 and ROUGE-L (Table S20) and (b) paired-bootstrap significance on per-sample ROUGE-1 differences (Table S21), both with 1,000 resamples over the 500 test samples. Three patterns emerge.

**Table 6.** MIMIC-IV clinical generation results (ROUGE %, 500 test samples per task). Boldface indicates the best result within each model size. The 2.7B block omits a base-only row because Mamba2+SFT is the appropriate fairness baseline at that scale. Per-arm 95% percentile-bootstrap CIs for R-1 and R-L at 2.7B (Table S20) and paired-bootstrap significance on per-sample ROUGE-1 differences at 2.7B (Table S21) are both in Section S14.

Model	Note completion			Discharge generation		
	R-1 ↑	R-2 ↑	R-L ↑	R-1 ↑	R-2 ↑	R-L ↑
*130M*						
Mamba2	4.63	0.80	3.43	4.83	0.35	3.56
Mamba2+SFT	6.84	2.36	4.95	8.79	2.76	6.30
BioMamba+SFT	**7.07**	**2.45**	**5.10**	**9.74**	**3.33**	**6.96**
*370M*						
Mamba2	5.04	0.97	3.69	4.50	0.33	3.31
Mamba2+SFT	7.85	3.04	5.62	8.96	2.69	6.19
BioMamba+SFT	**7.94**	**3.20**	**5.69**	**9.22**	**3.00**	**6.39**
*780M*						
Mamba2	5.46	1.17	3.86	5.22	0.37	3.59
Mamba2+SFT	**7.90**	**3.24**	**5.73**	**8.86**	**2.73**	**6.14**
BioMamba+SFT	**7.90**	**3.32**	**5.78**	**9.10**	**2.97**	**6.30**
*1.3B*						
Mamba2	5.61	1.28	3.95	5.33	0.42	3.68
Mamba2+SFT	7.93	3.28	5.76	9.99	**3.70**	**7.04**
BioMamba+SFT	**8.11**	**3.33**	**5.89**	**10.11**	**3.69**	**7.04**
*2.7B*						
Mamba2	5.55	1.19	3.85	7.04	0.80	4.71
Mamba2+SFT	7.16	2.45	4.98	8.87	2.76	6.21
BioMamba+SFT	**7.57**	**2.83**	**5.30**	**9.39**	**3.01**	**6.46**

CI, confidence interval; R-1/2/*L*, Rouge-1/2/*L*

First, clinical SFT is the dominant driver of generation quality: At the 130M, 370M, 780M, and 1.3B scales where base Mamba2 rows are reported, both Mamba2+SFT and BioMamba+SFT substantially outperform the corresponding base models on every ROUGE metric.

Second, BioMamba+SFT consistently matches or outperforms Mamba2+SFT on ROUGE-1 and ROUGE-L at every scale on both completion and discharge generation, with the single exception of a 0.01-point ROUGE-2 gap on 1.3B discharge (3.70 vs. 3.69). The advantage is most pronounced on discharge generation at 130M (ROUGE-1 9.74 vs. 8.79, Δ = +0.95). At 2.7B, paired-bootstrap tests on per-sample ROUGE-1 differences give $P\left(\Delta >0\right)=1.00$ for completion and $P\left(\Delta >0\right)=0.997$ for discharge, so the advantage is not within test-sample noise.

Third, within each arm, MIMIC-IV ROUGE improves with scale up to 1.3B (BioMamba+SFT reaches 8.11 / 10.11 on completion / discharge at 1.3B) and is slightly lower at 2.7B (7.57 / 9.39); the BioMamba+SFT advantage over Mamba2+SFT nonetheless persists at every scale, so the effect of continued pretraining is not absorbed by scaling on this dataset.

#### Representative case studies

The ROUGE improvements corresponded to clear qualitative differences in clinical generation. Table 7 shows a representative note-completion example from the 1.3B model family. The base Mamba2 model incorrectly propagated the cardiac abbreviation “RRR” to unrelated organ systems, producing clinically implausible output. Mamba2+SFT corrected the basic clinical register, whereas BioMamba+SFT produced the most complete continuation, including pertinent negatives and a natural transition to the discharge physical-examination section.

**Table 7.** Representative note-completion case study from the 1.3B model family. The input ends with “... HEART: RRR, no MRG, nl”. Each nonreference row contains a short excerpt from the model’s generation (before the em-dash) and a 1-line author reading (in italics) of that excerpt. Full raw outputs for this and 3 additional examples are in Section S6. Boldface indicates the best (highest) ROUGE score (the BioMamba+SFT row).

Source	Excerpt (before em-dash) + author reading (after em-dash)	R-1
Reference	S1-S2. LUNGS: CTA bilat, no ronchi/rales/wheezes... ABDOMEN: Midline surgical scar. Right sided PD catheter... EXTREMITIES: WWP, no edema, 2+ peripheral pulses. NEURO: Awake, A&Ox3, CNs II-XII grossly intact. *(reference text; no reading.)*	—
Mamba2	“LUNGS: RRR, no rales; ABDOMEN: RRR, no tenderness; URINARY: RRR... DIAPER: RRR” — *incorrectly applies the cardiac rhythm descriptor “RRR” to unrelated organ systems.*	2.73
Mamba2+SFT	“S1-S2; LUNGS: CTAB, no r/rh/wh... ABDOMEN: Soft, NTND... EXTREMITIES: WWP” — *clinically plausible continuation with standard abbreviations.*	3.65
**BioMamba+SFT**	“S1-S2; LUNGS: CTAB, no wheezes, rales, rhonchi... ABDOMEN: Soft, NTND, no rebound or guarding...NEURO: A&Ox3, moving all four extremities... Physical Exam on Discharge:” — *adds pertinent negatives and transitions to the discharge physical-examination section.*	**3.84**

Table 8 shows a representative discharge-summary generation example after vascular surgery. Here the base Mamba2 model largely repeated procedural details, Mamba2+SFT generated only a partial discharge framework, and BioMamba+SFT moved directly to a structured discharge-medication section with a clinically coherent regimen. These examples indicate that the quantitative gains reflect better completion and summarization behavior rather than only small changes in lexical overlap. Additional qualitative cases, including multipanel laboratory continuation, medication-reconciliation continuation, hospital course generation, and concise discharge instructions, are provided in Section S6.

**Table 8.** Representative discharge-summary generation case study from the 1.3B model family. Input: Service: SURGERY; Chief Complaint: Foot Ulcer; Procedure: Ultrasound-guided access to the left common femoral artery, selective catheterization of the right superficial femoral artery... Cell text is a short excerpt from the model’s generation (verbatim tokens in quotes) followed by an author reading (in italics). Full raw outputs for this and 3 additional examples are in Section S6. Boldface indicates the best (highest) ROUGE score (the BioMamba+SFT row).

Source	Excerpt + author reading (italicized)	R-1	R-2
Reference	DISCHARGE MEDICATIONS: 1. Clopidogrel 75 mg PO DAILY 2. Sulfameth/Trimethoprim DS 2 TAB PO BID... *(reference text; no reading.)*	—	—
Mamba2	“8 x 80 mm Innova stent post dilated with a 7 mm balloon” (procedure phrasing repeated) — *repeats procedure details and fails to transition to discharge content.*	10.85	0.68
Mamba2+SFT	*Generates a partial discharge framework and follow-up surgical description, but no explicit medication list. (Excerpt omitted here for space; see Supplementary Section S6.)*	17.87	2.08
**BioMamba+SFT**	“DISCHARGE MEDICATIONS: Aspirin 81 mg, Atorvastatin 40 mg, Metoprolol Tartrate 50 mg...” — *concisely summarizes the procedure and moves directly to a structured medication regimen consistent with post-vascular surgery care.*	**23.05**	**3.75**

These results show that biomedical knowledge acquired during PubMed continued pretraining transfers to clinical text generation. The consistent advantage of BioMamba+SFT over Mamba2+SFT suggests that literature-based continued pretraining adds value even when clinical SFT data are available, although learning-rate tuning remains important during fine-tuning.

#### Output diagnostics

To complement the ROUGE comparison with quantitative summaries of generation behavior, we computed standard output diagnostics on 1.3B discharge generation under nucleus-0.9 and nucleus-0.95 decoding (Table 9): average length, distinct-1 and distinct-2 token-type ratios, and a repeated-4-gram rate. Greedy decoding remains the apples-to-apples primary setting for Table 6; we report diversity and repetition diagnostics only for the higher-entropy nucleus settings, where these metrics are informative. Under nucleus-0.95, a standard deployment setting for open-ended clinical text, BioMamba has slightly better diversity and a lower repeated-4-gram rate than Mamba2; under nucleus-0.9, the 2 arms are close. A lightweight section-header F1 structure metric is reported in Table S19.

**Table 9.** MIMIC-IV discharge output diagnostics at 1.3B under nucleus-0.9 and nucleus-0.95 decoding: average generated length, distinct-1 and distinct-2 token-type ratios, and the repeated-4-gram rate. Greedy decoding used in Table 6 is omitted from this table. Boldface indicates the better value between the two arms under nucleus-0.95 (higher distinct-1/2 and lower repeated-4-gram rate).

Model	Decoding	Avg length	Dist-1	Dist-2	Rep-4-gram
Mamba2+SFT	nucleus-0.9	74.6	0.730	0.876	0.071
BioMamba+SFT	nucleus-0.9	74.8	0.719	0.865	0.077
Mamba2+SFT	nucleus-0.95	74.1	0.770	0.914	0.040
**BioMamba+SFT**	nucleus-0.95	74.6	**0.777**	**0.918**	**0.036**

#### Factuality proxy analysis

To move beyond ROUGE as a surface-lexical metric and provide a grounding-oriented check of clinical generation, we added an natural language inference (NLI)-ensemble factuality proxy on 2.7B MIMIC discharge generations (Table 10). Using 2 Multi-Genre Natural Language Inference checkpoints (roberta-large-mnli and microsoft/deberta-v2-xlarge-mnli), a generation is flagged as contradictory only when both checkpoints independently output a contradiction probability above 0.7. We scored entailment of each generated discharge section against the input admission text only. Across all 6 tested cells, the contradiction rate lies in the 7.0% to 9.4% range, with Wilson 95% confidence intervals overlapping substantially between arms at every decoding. Macro-averaged across the 3 decoding strategies, BioMamba (7.4%) is below Mamba2 (8.1%); under nucleus-0.95 sampling, Mamba2 rises to 9.4% while BioMamba stays at 7.0%—a 2.4-pp gap in BioMamba’s favor. Continued pretraining therefore does not introduce systematic source-contradictory output relative to the base model and is more stable than the base model under higher-entropy sampling. We present this analysis as a factuality proxy rather than a clinician audit and treat safety, calibration, and workflow readiness as out-of-scope for this study (see Discussion); the NLI protocol and the schema of the autoflagged clinician-review candidate list are in Section S12.

**Table 10.** NLI-ensemble factuality proxy on 2.7B MIMIC discharge generation (*n* = 500 per cell; 2 MNLI checkpoints, roberta-large-mnli and microsoft/deberta-v2-xlarge-mnli). Contradiction rate is computed against the input admission text only; lower is better. Ninety-five perent confidence intervals are Wilson score intervals.

2.7B arm	Decoding	Contradictions / *n*	Rate (↓) [Wilson 95% CI]
M2+SFT	greedy	35 / 500	7.0% [5.1, 9.6]
	nucleus-0.9	39 / 500	7.8% [5.8, 10.5]
	nucleus-0.95	47 / 500	9.4% [7.1, 12.3]
	macro-avg	—	8.1%
BM+SFT	greedy	37 / 500	7.4% [5.4, 10.0]
	nucleus-0.9	39 / 500	7.8% [5.8, 10.5]
	nucleus-0.95	35 / 500	7.0% [5.1, 9.6]
	macro-avg	—	7.4%

CI, confidence interval; M2+SFT, Mamba2+SFT; BM+SFT, BioMamba+SFT

#### Failure modes

The remaining errors are consistent with the behaviors already visible in the qualitative examples. For MIMIC, the main residual issues are occasional repetition under greedy decoding, occasional truncation of the target section when the 128-token budget is reached before the discharge medication list completes, and incomplete coverage of medication-level detail in a subset of generations; these are directly reflected in the nucleus-0.95 diagnostic improvements and in the qualitative case studies in Section S6. For the question-answering tasks, most BioASQ errors at larger scales are shared between Mamba2+SFT and BioMamba+SFT: At 780M, every item BioMamba+SFT gets wrong is also missed by Mamba2+SFT, and at 1.3B, the overlap is 11 of 12 (see Section S11). Residual disagreements at larger scales are therefore confined to a handful of genuinely hard items rather than indicating a regression attributable to continued pretraining.

### Contextual comparison with external models on MIMIC-IV

Table 11 compares BioMamba’s clinical generation results with 7 public biomedical or medically adapted language models on the same 500 MIMIC-IV test samples under identical greedy decoding. The external baselines were not given MIMIC-specific SFT, so this is a contextual rather than task-matched comparison; caveats are summarized in the caption.

**Table 11.** Comparison of BioMamba with external biomedical and medically adapted language models on MIMIC-IV clinical generation (ROUGE %, 500 test samples)

Model	Architecture	Parameters	Note completion			Discharge generation		
			R-1 ↑	R-2 ↑	R-L ↑	R-1 ↑	R-2 ↑	R-L ↑
BioGPT	GPT-2	347M	1.20	0.16	1.01	1.53	0.09	1.26
BioMedLM	GPT-2	2.7B	1.58	0.16	1.17	2.18	0.13	1.59
BioGPT-Large	GPT-2	1.5B	3.85	0.54	2.81	3.20	0.20	2.42
Gemma3-1B (fine-tuned)	Gemma3	1B	4.18	0.65	2.83	5.55	0.42	3.77
BioGPT-Large-PubMedQA	GPT-2	1.5B	4.75	0.75	3.04	5.28	0.41	3.30
Bio-Medical-Llama-3.2-1B	Llama 3.2	1B	4.80	0.82	3.19	5.58	0.43	3.70
Meditron3-Gemma2-2B	Gemma2	2B	5.13	0.89	3.37	6.04	0.55	3.88
BioMamba-130M+SFT	Mamba2	130M	7.07	2.45	5.10	9.74	3.33	6.96
BioMamba-1.3B+SFT	Mamba2	1.3B	8.11	3.33	5.89	10.11	3.69	7.04

PubMedQA, PubMed Question Answering; R-1/2/*L*, Rouge-1/2/*L*

Within this contextual setup, the task-specific BioMamba rows lie above the untuned external baselines on both MIMIC tasks. We interpret this only as evidence that BioMamba is competitive relative to released checkpoints in the current landscape, not as a controlled superiority claim, because the comparison does not isolate architecture, tokenizer, pretraining data, or task-adaptation protocol. The observed gap should therefore be read descriptively rather than causally.

### Biomedical question answering on BioASQ

BioASQ yes/no question answering complements the MIMIC experiments because it tests literature-oriented biomedical reasoning rather than clinical document generation. Downstream performance after SFT, together with the corresponding Mamba2+SFT baseline accuracies and gains, is summarized in Table 12. BioASQ gains from continued pretraining are scale-dependent and nonmonotonic: The 370M row is slightly below the Mamba2+SFT baseline, 130M, 780M, and 1.3B are positive, and 2.7B ties the baseline. Because the BioASQ yes/no test split contains only 82 items, a single-question difference corresponds to 1.22 pp, which matches the magnitude of the 370M and 2.7B deltas; a 3-seed reproduction across all 5 scales (seeds {13,42,97}; Table S16) gives σ≤0.021 on BioASQ and σ≤0.032 on PubMedQA, and an item-level error analysis (Table S18) shows that BioMamba fixes more BioASQ items than it breaks at every scale while most residual errors are shared between the 2 arms. In contrast to the nonmonotonic BioASQ pattern, PubMedQA gains from continued pretraining are positive at every scale. Within the BioMamba series itself, performance improved overall with model scale: BioASQ accuracy increased from 68.29% at 130M to 90.24% at 2.7B (Fig. 3), and the macro-F1 score increased from 0.634 to 0.890. The strongest model, BioMamba-2.7B, achieved 90.24% accuracy (74/82, 95% Wilson confidence interval, 81.9% to 95.0%) and a macro-F1 score of 0.890. Together with the internal perplexity and MIMIC findings, these results indicate that gains from biomedical language modeling transfer across biomedical literature and clinical text rather than remaining confined to perplexity.

**Table 12.** Downstream question-answering results across model scales. Each M2+SFT and BM+SFT cell reports raw correct/total counts together with accuracy. Test-set sizes: BioASQ *n* = 82; PubMedQA *n* = 200. The corresponding 95% Wilson binomial confidence intervals for the BM+SFT cells are in Table S17; 3-seed reproduction means ± SDs are in Table S16. Boldface indicates the highest accuracy and macro-F1 in each column (BioMamba-2.7B).

Scale	BioASQ				PubMedQA			
	M2+SFT	BM+SFT	Gain	F1	M2+SFT	BM+SFT	Gain	F1
130M	44/82 (53.66%)	56/82 (68.29%)	+14.63	0.634	112/200 (56.00%)	126/200 (63.00%)	+7.00	0.661
370M	65/82 (79.27%)	64/82 (78.05%)	−1.22	0.721	131/200 (65.50%)	132/200 (66.00%)	+0.50	0.690
780M	64/82 (78.05%)	65/82 (79.27%)	+1.22	0.778	129/200 (64.50%)	135/200 (67.50%)	+3.00	0.711
1.3B	68/82 (82.93%)	70/82 (85.37%)	+2.44	0.831	130/200 (65.00%)	140/200 (70.00%)	+5.00	0.740
2.7B	74/82 (90.24%)	**74/82 (90.24%)**	+0.00	**0.890**	141/200 (70.50%)	**146/200 (73.00%)**	+2.50	**0.773**

BioASQ, Biomedical Semantic Indexing and Question Answering; PubMedQA, PubMed Question Answering; M2+SFT, Mamba2+SFT; BM+SFT, BioMamba+SFT; Gain, BM+SFT - M2+SFT in pp; F1, BM+SFT macro-F1

**Fig. 3.**
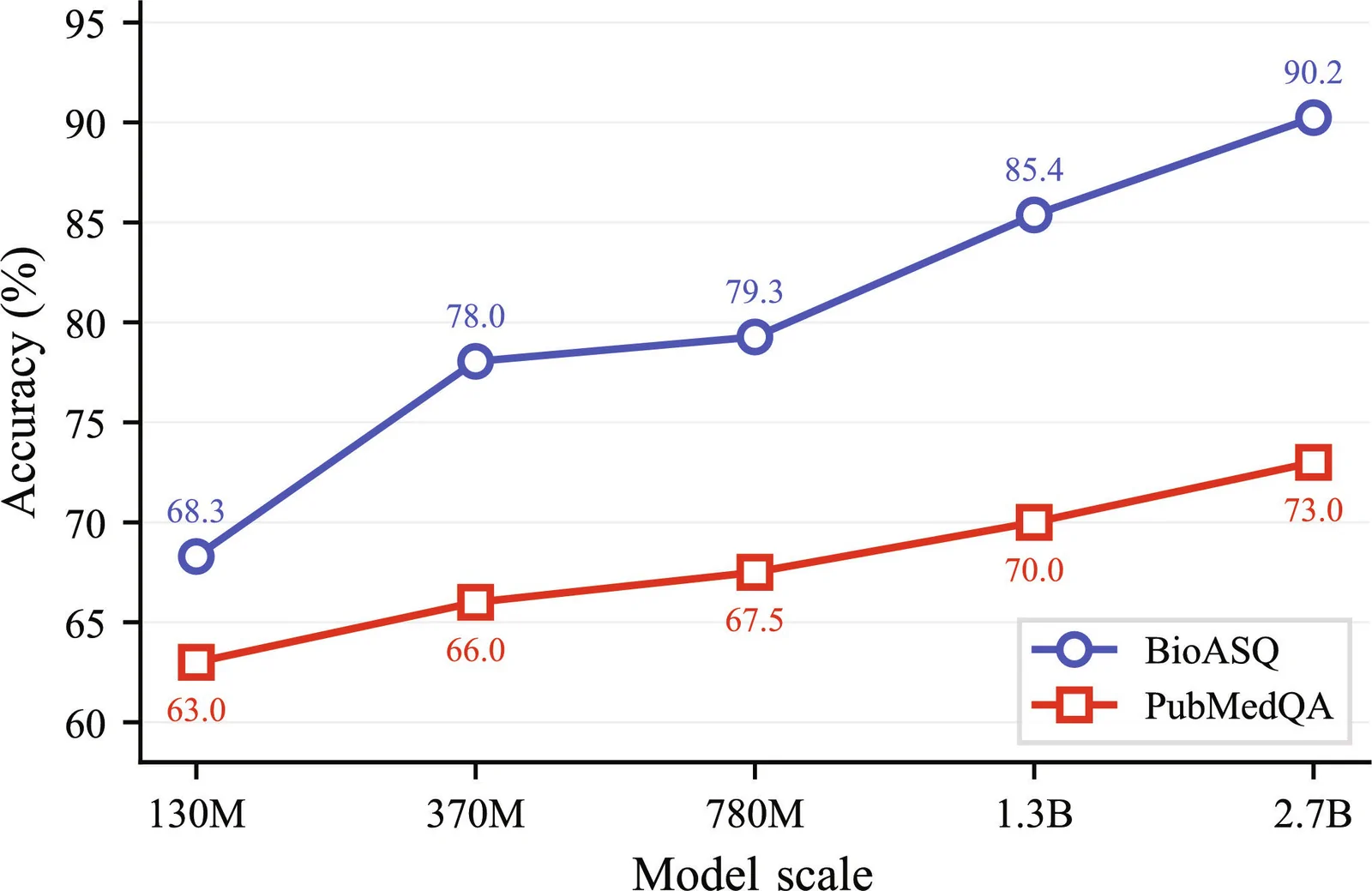
Downstream question-answering accuracy as a function of model scale. Both Biomedical Semantic Indexing and Question Answering (BioASQ) and PubMed Question Answering (PubMedQA) accuracy improve consistently from 130M to 2.7B parameters.

### Biomedical question answering on PubMedQA

Binary PubMedQA was the third evaluation task. Relative to the corresponding Mamba2+SFT baselines, BioMamba improved PubMedQA accuracy at every scale, and performance also improved overall with model scale within the BioMamba series (Table 12). PubMedQA accuracy increased from 63.00% at 130M to 73.00% at 2.7B, and the macro-F1 score increased from 0.661 to 0.773. The strongest model, BioMamba-2.7B, achieved 73.00% accuracy (146/200; 95% confidence interval, 66.5% to 78.7%) and a macro-F1 score of 0.773.

### Ablation studies

#### Data-mixture ablation

The first ablation examined the composition of the continued pretraining corpus. As shown in Table 13 and Fig. 4, PubMed-only continued pretraining improved in-domain perplexity but worsened both Wikipedia and C4 perplexity, consistent with forgetting. Most mixed-corpus settings retained the PubMed gain, but many still weakened at least 1 held-out benchmark. The selected 10% C4 + 10% Wikipedia setting was the most balanced configuration: It achieved the lowest PubMed perplexity, the largest Wikipedia improvement, and essentially unchanged C4 perplexity. These results support the final 80% PubMed, 10% C4, and 10% Wikipedia mixture used for the main BioMamba checkpoints. A complementary 370M PubMed-only control, reported in Table S13, confirms that this mixture remains load-bearing at a larger scale: PubMed-only continued pretraining worsens Wikipedia-style held-out perplexity by 69% and C4 perplexity by 15% and is also slightly worse on held-out PubMed itself (9.32 → 9.91), consistent with the 130M pilot where the 80%/10%/10% mixture already matched PubMed-only on PubMed (8.41 vs. 8.47); the gap is larger at 370M, indicating that domain-only continued pretraining incurs a larger forgetting penalty at scale even on in-domain text.

**Table 13.** Full continued pretraining data-mixing ablation for the 130M pilot selection run. Negative Δ values indicate lower perplexity than the base model.

Configuration	C4 (%)	Wikipedia (%)	PubMed MML	PubMed Δ	Wikipedia PPL	Wikipedia Δ	C4 PPL	C4 Δ
No continued pretraining	0	0	9.39	—	23.29	—	26.51	—
PubMed only	0	0	8.47	↓9.8%	24.50	↑5.2%	27.90	**↑**5.2%
C4 only, 5%	5	0	8.48	↓9.7%	24.03	↑3.2%	26.53	**↑**0.1%
C4 only, 10%	10	0	8.99	↓4.2%	25.28	↑8.5%	27.80	**↑**4.9%
C4 only, 20%	20	0	8.48	↓9.7%	24.19	↑3.9%	26.34	↓0.7%
C4 only, 30%	30	0	8.49	↓9.6%	24.25	↑4.1%	26.29	↓0.8%
Wikipedia only, 10%	0	10	8.48	↓9.7%	23.79	↑2.2%	27.07	**↑**2.1%
Wikipedia only, 20%	0	20	8.49	↓9.5%	23.83	↑2.3%	27.07	**↑**2.1%
Wikipedia only, 30%	0	30	8.50	↓9.5%	23.84	↑2.4%	27.08	**↑**2.2%
5% C4 + 5% Wikipedia	5	5	8.48	↓9.7%	23.75	↑2.0%	26.50	↓0.0%
5% C4 + 10% Wikipedia	5	10	8.49	↓9.6%	23.76	↑2.0%	26.50	↓0.0%
10% C4 + 5% Wikipedia	10	5	8.49	↓9.6%	23.78	↑2.1%	26.42	↓0.3%
10% C4 + 10% Wikipedia	10	10	8.41	↓10.4%	18.95	↑18.6%	26.51	0.0%
10% C4 + 20% Wikipedia	10	20	8.50	↓9.5%	23.76	↑2.0%	26.45	↓0.2%
15% C4 + 15% Wikipedia	15	15	8.50	↓9.5%	23.77	↑2.1%	26.39	↓0.4%
20% C4 + 10% Wikipedia	20	10	8.49	↓9.5%	23.75	↑2.0%	26.34	↓0.6%
20% C4 + 20% Wikipedia	20	20	8.50	↓9.5%	23.70	↑1.8%	26.36	↓0.6%

C4, Colossal Clean Crawled Corpus; PPL, perplexity

**Fig. 4.**
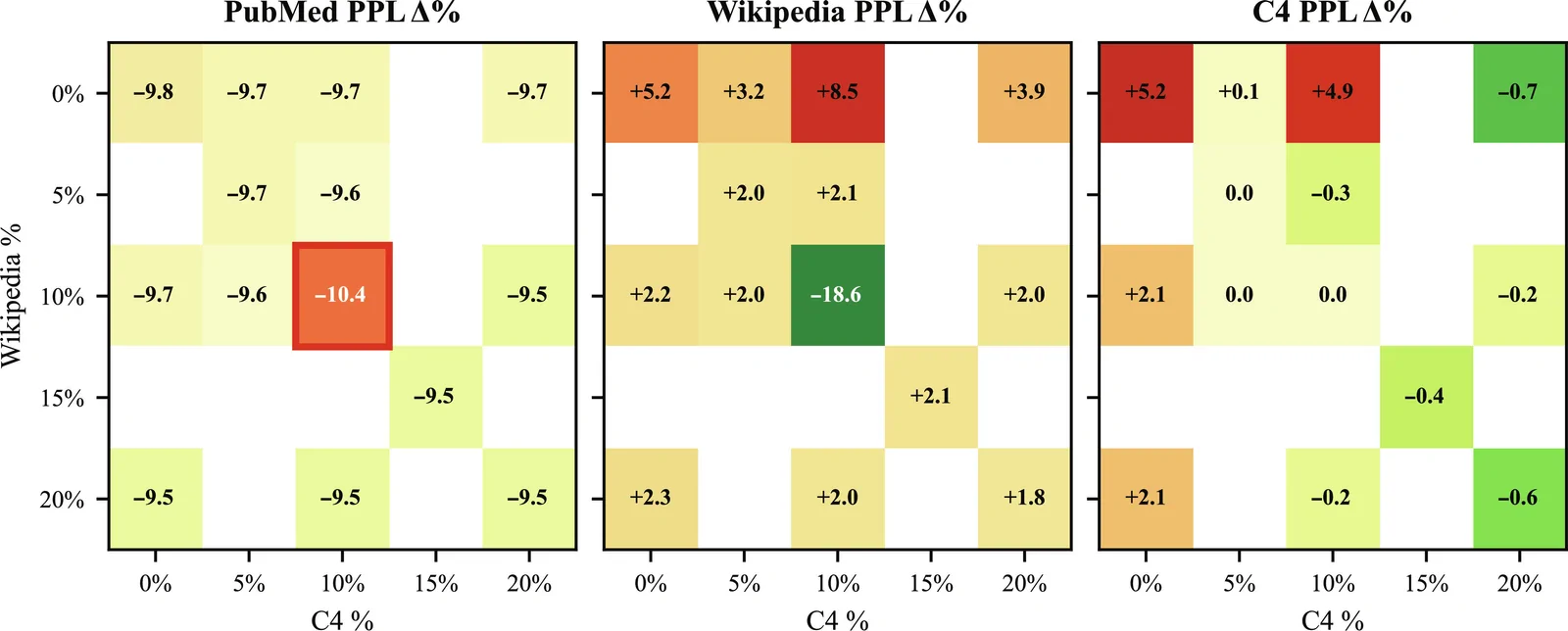
Data-mixture ablation heatmap showing the perplexity change (%) relative to the base model for PubMed, Wikipedia, and Colossal Clean Crawled Corpus (C4). Each cell corresponds to a C4/Wikipedia mixture ratio; the red box highlights the selected 10%/10% configuration.

#### Continued pretraining ablation before SFT

The second ablation examined the role of continued pretraining before SFT. At 130M (Table 12), continued pretraining increased BioASQ accuracy from 53.66% (44/82; 95% confidence interval, 42.9% to 64.0%) to 68.29% (56/82; 95% confidence interval, 57.6% to 77.4%), with macro-F1 rising from 0.536 to 0.634; PubMedQA accuracy increased from 56.00% (112/200; 95% confidence interval, 49.1% to 62.7%) to 63.00% (126/200; 95% confidence interval, 56.1% to 69.4%), with macro-F1 from 0.570 to 0.661. The class-specific pattern differs between benchmarks: On BioASQ, the yes F1 rose from 0.513 to 0.768 while the no F1 dropped from 0.558 to 0.500, indicating that in the smallest model, biomedical pretraining mainly strengthened recognition of affirmative evidence patterns; on PubMedQA, both yes and no F1 improved (0.667→0.727 and 0.472→0.595), consistent with a more symmetric benefit (Table S6).

Across scales (Table 12), on PubMedQA continued pretraining improved accuracy at every scale, with gains ranging from 0.50 to 7.00 pp. On BioASQ, the gain was large at 130M, modest at 780M and 1.3B, neutral at 2.7B, and slightly negative at 370M. These results show that the value of biomedical continued pretraining after task-specific supervision depends on both model scale and downstream task.

### Learning curves and convergence

Optimization remained stable across all scales (Fig. 5): Validation loss and perplexity decreased smoothly during continued pretraining, and the best validation point occurred at the final step in every run (lowest validation losses: 2.11 / 1.90 / 1.80 / 1.72 / 1.65 at 130M / 370M / 780M / 1.3B / 2.7B). These patterns indicate that the 3-epoch schedule was well calibrated and that the reported language-model improvements are unlikely to be artifacts of unstable optimization or selective early stopping.

**Fig. 5.**
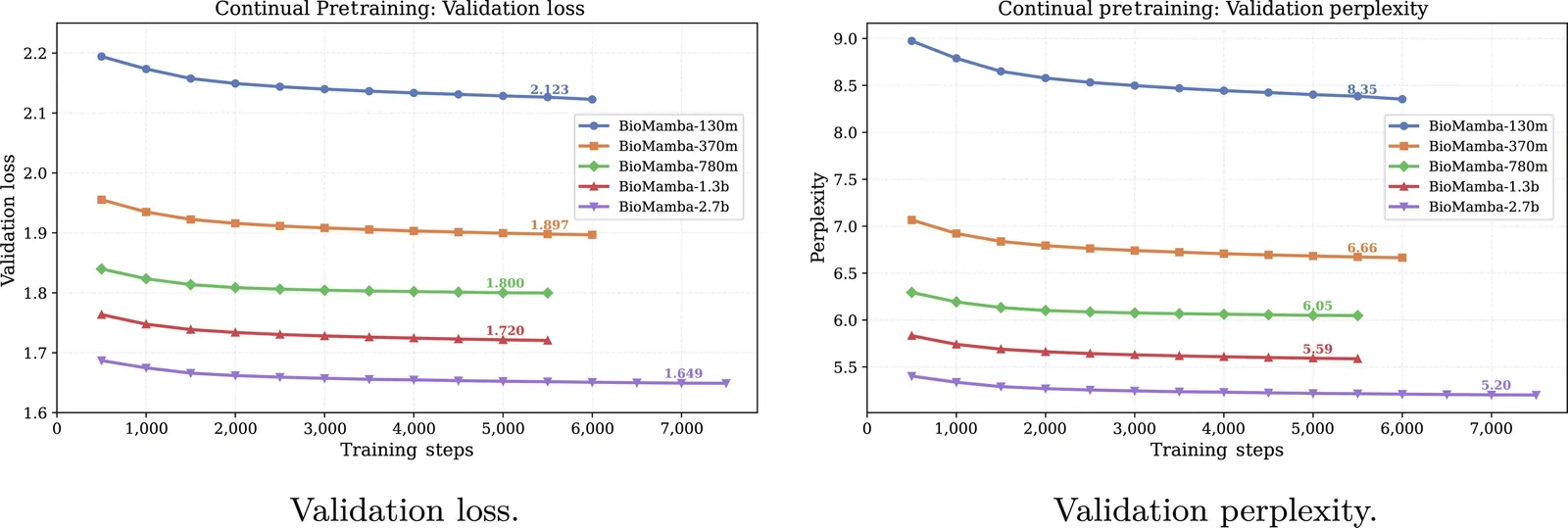
Training dynamics during continued pretraining. All model scales converged smoothly, and the best validation point occurred at the final step of every run. The 3-epoch continued pretraining budget was chosen because validation loss had effectively plateaued by the end of training; additional training epochs produced diminishing reductions in validation loss, so the 3-epoch schedule is convergence-driven rather than a fixed-budget compromise.

## Discussion

This study examined whether biomedical continued pretraining can improve Mamba language models without substantially weakening general-language-modeling fluency. The results support this goal across both biomedical literature and clinical text. Under tokenizer-controlled internal evaluation, BioMamba lowered PubMed perplexity at every model scale, improved Wikipedia-style held-out perplexity, and left C4 performance largely unchanged; a 6-task out-of-domain multiple-choice suite and an evaluation-budget stability check (“Out-of-domain evaluation and evaluation-budget stability” section) further support preservation on the benchmarks tested. After SFT, the models also transferred across 3 downstream tasks: note completion, discharge summary generation, and biomedical yes/no question answering. Taken together, the findings support balanced domain adaptation as a practical strategy for biomedical state space language models.

### Practical biomedical and clinical applications

The most immediate use of BioMamba is workflow support rather than autonomous bedside decision-making. The PubMed, BioASQ, and PubMedQA results are most relevant to tasks such as literature screening, evidence extraction, abstract triage, biomedical question answering, and drafting structured evidence summaries [48,49]. The MIMIC-IV results extend this picture to clinical documentation support, especially note completion and discharge summary generation, which are closely related to hospital-course summarization and discharge-note generation tasks studied previously [36–39,50]. These tasks require models to handle biomedical terminology together with broader scientific or clinical language, which fits the balanced adaptation strategy used here. Recent studies of medical large language models report strong performance on clinical knowledge and medical question answering benchmarks [51–54], but discussions of clinical deployment similarly suggest that the most credible near-term role of such models is workflow support with retrieval and human review rather than autonomous clinical decision-making [55,56].

The scale analysis also has practical implications. Continued pretraining produced its largest downstream gains in the smaller models, suggesting that useful biomedical adaptation is not limited to the largest checkpoint. This is relevant for institutions that require lower inference cost, local deployment, or more reproducible fine-tuning under limited computational budgets. At the same time, the present results do not justify direct use for patient-specific diagnosis, prognosis, or treatment recommendation. BioMamba was trained mainly on PubMed-centered text and evaluated on benchmark question answering and clinical generation rather than on prospective clinical tasks. Its most suitable role is therefore as an assistive component in literature-grounded or documentation-support pipelines with retrieval, source attribution, and expert oversight [55,57].

### Related work

Biomedical language modeling has been dominated by Transformer-based architectures, including SciBERT [3], BioBERT [2], PubMedBERT [4], BioGPT [5], SciFive [6], BioBART [7], and BioMedLM [21], which established the value of domain-specific pretraining for biomedical natural language processing. Text-to-text pretraining paradigms such as T5 [58] and link-aware literature pretraining approaches such as LinkBERT [59] further expanded the design space for biomedical language modeling. Clinical-domain models such as ClinicalBERT [8], GatorTron [9], ClinicalT5 [10], and broader electronic health record-oriented language models [60,61] extended domain adaptation to clinical notes [62]. Work on hospital-course summarization and clinical text generation has also expanded, including dataset construction, model adaptation, and shared-task evaluation [36–40,50]. Biomedical question answering has a mature benchmark ecosystem centered on BioASQ [41–45] and PubMedQA [46]. More recent open medical language models such as PMC-LLaMA [22], BioMistral [23], MEDITRON-70B [24], and Clinical Camel [63] still rely mainly on Transformer-style backbones. In parallel, structured state space models [18], Mamba [19], and state space duality refinements [20] have provided an alternative framework with favorable scaling for long inputs [64]. However, biomedical adaptation of these models remains limited, and published work has so far focused more narrowly on settings such as clinical text modeling [25].

The present study differs from much of this literature in 2 ways. First, the main evidence is based on tokenizer-controlled comparisons between each released base checkpoint and the corresponding checkpoint after continued pretraining. This design isolates the effect of biomedical adaptation more directly than comparisons across unrelated public models. Second, the adaptation strategy explicitly addresses the trade-off between biomedical specialization and retention of general-language-modeling fluency. That trade-off matters in biomedical literature workflows, where specialized terminology is often mixed with broader scientific language, and it has been emphasized in continued pretraining studies [26–28]. The question also relates to ongoing debate about whether generalist foundation models can replace specialized biomedical or clinical models [65,66]. Recent benchmark studies in biomedical NLP and real-world clinical practice text [67,68], together with cautionary few-shot results in the biomedical domain [69], suggest that conclusions depend strongly on task formulation and evaluation protocol. Recent methodological work has also argued that benchmark gains should be interpreted cautiously and connected to realistic biomedical use settings [57]. In that context, the present study aims to provide controlled and reproducible evidence on biomedical continued pretraining for Mamba-family models rather than to claim clinical readiness.

### New findings

Several findings merit emphasis.

First, the mixture ratio mattered more than the raw inclusion of nonbiomedical data. At 130M, PubMed-only continued pretraining worsened Wikipedia-style held-out perplexity by 5.2% and C4 perplexity by 5.2% relative to the base model, and most alternative mixtures we tested retained the PubMed gain but still weakened at least 1 held-out benchmark. At 370M, the PubMed-only control was worse than the 80%/10%/10% recipe on Wikipedia (+69%), C4 (+15%), and even held-out PubMed itself (9.32 → 9.91; Table S13), indicating that the forgetting penalty grows with scale and that adding general-domain data did not trade off against in-domain performance. The 80%/10%/10% recipe therefore appears effective because it strikes a specific balance rather than because it simply adds more data, and this balance is load-bearing beyond the original 130M pilot.

Second, the downstream effect of continued pretraining after SFT was jointly task- and scale-dependent: PubMedQA gains were positive at every scale, MIMIC-IV gains were positive at every scale on both completion and discharge generation, while BioASQ gains were nonmonotonic and the 370M and 2.7B deltas fell within the 1.22-pp single-question resolution and the seed-level variance reported in Table S16. A likely explanation is that smaller models benefit more from additional domain exposure, whereas larger models may already contain enough transferable knowledge for some supervised tasks—yet biomedical adaptation and scaling remained complementary, since the BioMamba+SFT advantage over Mamba2+SFT persisted at every evaluated scale even where absolute ROUGE plateaued at 1.3B on MIMIC-IV.

### Limitations

Several limitations should be considered when interpreting these results. The continued pretraining corpus was restricted to PubMed abstracts and did not include full-text biomedical articles, clinical narratives, electronic health records, or multimodal clinical data [70]. The downstream evaluation, although broader than yes/no question answering alone, remained limited to 3 task families—note completion, discharge summary generation, and yes/no question answering—and therefore does not cover the full range of biomedical language model utility. Additional tasks such as information extraction, retrieval, evidence attribution, broader summarization, and long-context reasoning were not evaluated, despite their prominence in recent biomedical and clinical benchmark suites [67,68].

The mixture-ratio search was performed primarily in a 130M pilot study, and the selected mixture was then applied across all model scales, with a single 370M cross-scale ablation confirming that the mixture is load-bearing at larger scales. Although the cross-scale results were broadly consistent, this design does not exclude the possibility that different scales would benefit from different data proportions or training schedules.

Our comparisons with publicly released biomedical checkpoints (Tables 3 and 11) are contextual rather than controlled, as discussed in Methods and Results, and should not be read as leaderboard-style performance claims. In addition, the benchmark datasets were modest in size, especially the BioASQ yes/no evaluation set, so small absolute differences should be interpreted cautiously.

A further limitation is that the study did not assess calibration, uncertainty estimation, hallucination behavior, robustness to distribution shift, or safety in real clinical workflows [71]. Our factuality analysis (Results) uses NLI contradiction as a proxy for source faithfulness; this does not replace a clinician audit, and the current study does not establish clinical safety, uncertainty calibration, or workflow readiness. A prospective clinician audit with a structured error taxonomy is left to future work. The evaluation scope of this study is in-domain biomedical QA and clinical generation, together with general-language-modeling fluency; broader capabilities such as retrieval-grounded reasoning, evidence-grounded citation, structured information extraction, long-context clinical reasoning, and prospective deployment were not evaluated and are therefore hypothesized rather than demonstrated. Improvement on benchmark question answering or clinical generation does not by itself establish clinical validity or deployment readiness [57]. The present results should therefore be interpreted as evidence about language-model adaptation and transfer, not as evidence that BioMamba is suitable for autonomous clinical use.

### Future work

Several directions follow directly from these limitations. An immediate next step is to expand the continued pretraining corpus to include full-text biomedical articles, clinical guidelines, trial reports, and carefully governed clinical narratives when available. It would also be useful to study whether the optimal PubMed/C4/Wikipedia balance changes with model size, training budget, or target task. More systematic ablations on data mixture, training duration, and learning-rate schedules may further refine the adaptation strategy.

Future evaluation should extend beyond the present tasks of note completion, discharge summary generation, and yes/no question answering to tasks that are closer to biomedical and clinical workflows [72], including retrieval-augmented question answering, evidence extraction, literature screening, broader summarization, uncertainty estimation, and source-grounded generation [55,56]. It will also be important to assess robustness, calibration, and error patterns in settings with expert review. More broadly, the present results suggest that biomedical Mamba models may be most useful as components in retrieval-grounded and clinician-supervised systems rather than as standalone models. Testing that hypothesis in end-to-end health data science pipelines is an important next step.

## Conclusion

BioMamba shows that biomedical domain-adaptive continued pretraining can strengthen public Mamba2 checkpoints without changing the underlying architecture. Across 5 model scales from 130M to 2.7B, BioMamba improves biomedical language modeling, preserves general-language-modeling fluency through a balanced 80%/10%/10% PubMed/C4/Wikipedia training corpus, and transfers effectively to 3 downstream tasks: clinical note completion, discharge summary generation, and biomedical question answering. Beyond the empirical findings, our main contribution is a reproducible biomedical continued pretraining recipe together with a family of open-weight BioMamba checkpoints (130M / 370M / 780M / 1.3B / 2.7B), released publicly with the full training and evaluation code to provide the biomedical natural language processing community with a reusable resource.

## Ethical Approval

No new human-subject data were collected. This study used public text corpora and benchmark datasets together with deidentified clinical notes from MIMIC-IV-Note v2.2, accessed under PhysioNet’s credentialed data use terms. All figures in this manuscript were created by the authors, who hold the copyright.

## Data Availability

Training and evaluation code, configurations, and the 5 BioMamba checkpoints (130M / 370M / 780M / 1.3B / 2.7B) are publicly available at https://github.com/LeoYML/BioMamba; the released checkpoints are also collected at https://huggingface.co/collections/zmzfpc/biomamba. The release is under the Apache-2.0 license, matching the upstream Mamba2 checkpoints. The continued pretraining corpora were loaded from Hugging Face as cyrilzakka/pubmed-medline (default, revision 432681e19469e93e6c42878d5f41fec400974fb8), wikimedia/wikipedia (20231101.en, revision b04c8d1ceb2f5cd4588862100d08de323dccfbaa), and allenai/c4 (en, revision 1588ec454efa1a09f29cd18ddd04fe05fc8653a2); the Wikipedia-style held-out evaluation set uses the Wikitext-103 raw split, as described in Section S15. BioASQ and PubMedQA are publicly available from their original sources. MIMIC-IV-Note v2.2 is available to credentialed users through PhysioNet under its data-use requirements. Public checkpoint identifiers used for the contextual comparisons are listed in Section S3, and additional reproducibility details (corpus snapshots, held-out construction, preprocessing rules, prompt templates, and model-selection criteria) are provided in Section S15.

## Supplementary Materials

Supplementary 1 Supplementary Section S1 to S15 Tables S1 to S21 Fig. S1
