# Supplementary material for: BioMamba: Domain-Adaptive Biomedical Language Models
Source: Health Data Sci. 2026 Oct 5;6:0454. doi: 10.34133/hds.0454 (PMC13635941; doi:10.34133/hds.0454)
Supplement: Supplementary file 1 [file hds.0454.f1.docx]

# Supplementary Materials

This supplement provides implementation details, evaluation details, exact public checkpoint sources, class-specific downstream results, additional clinical generation case studies, and supplementary tables covering the cross-scale mixture ablation, out-of-domain evaluation and evaluation-budget stability, matched-budget SFT control, 3-seed QA reproduction, BioASQ item-level error analysis, NLI factuality protocol, MIMIC output diagnostics, bootstrap confidence intervals, and reproducibility details.

# S1 Experimental Environment

All continued pretraining and language-model evaluation experiments were conducted in the environment summarized in Table S1.

Table S1: Experimental environment for continued pretraining and evaluation.

| **Item** | **Version / Specification** |
| --- | --- |
| GPU | NVIDIA H100 80 GB HBM3 |
| GPU driver | 580.82.07 |
| CUDA | 12.8 |
| Operating system | Ubuntu 24.04.1 LTS (Kernel 5.14.0) |
| Python | 3.12.3 |
| PyTorch | 2.10.0+cu128 |
| mamba-ssm | 2.3.0 |
| transformers | 4.57.1 |
| datasets | 4.0.0 |

# S2 Continued Pretraining Implementation Details

Table S2 summarizes the hyperparameters shared across BioMamba continued pretraining runs, and Table S3 provides per-scale implementation details.

Table S2: Shared continued pretraining hyperparameters.

| **Parameter** | **Value** | **Description** |
| --- | --- | --- |
| Epochs | 3 | Training epochs |
| Weight decay | 0.1 | AdamW weight decay |
| Maximum sequence length | 1,024 | Tokens per sequence |
| Gradient clipping | 1.0 | Maximum gradient norm |
| Precision | bfloat16 | Mixed precision training |
| Seed | 42 | Random seed |
| *Warmup-stable-decay scheduler*  Warmup ratio | 0.10 | Linear warmup |
| Stable ratio | 0.65 | Constant learning-rate plateau |
| Decay ratio | 0.20 | Cosine decay |
| Minimum learning-rate ratio | 0.1 | Minimum learning-rate ratio |
| *Advanced techniques*  Exponential moving average | Enabled | Decay = 0.999 |
| Layer-wise learning-rate decay | 0.90–0.95 | Lower layers adapt more conservatively |

Table S3: Per-scale continued pretraining configuration, combining optimization (batch, gradient accumulation, effective batch size, layer-wise learning-rate decay, maximum learning rate, total run steps) and resource usage (number of GPUs, peak memory per GPU). The base learning rate was set conservatively and scaled by model size to reduce catastrophic forgetting during continued

pretraining.

| **Scale** | **Batch/GPU Accum** | **Eff. BS** | **LR decay** | **Max. LR** | **Steps** | **# GPUs** | **Peak mem.** |
| --- | --- | --- | --- | --- | --- | --- | --- |
| 130M | 32 8 | 256 | 0.90 | 1.5e-6 | 5,967 | 1*×* H100 | *∼*40 GB |
| 370M | 48 5 | 240 | 0.95 | 4.5e-7 | 6,363 | 1*×* H100 | *∼*76 GB |
| 780M | 32 8 | 256 | 0.95 | 3.0e-7 | 5,967 | 1*×* H100 | *∼*65 GB |
| 1.3B | 16 16 | 256 | 0.95 | 2.5e-7 | 5,967 | 1*×* H100 | *∼*65 GB |
| 2.7B | 6 8 | 192 | 0.95 | 2.0e-7 | 7,953 | 4*×* H100 | *∼*80 GB |

# S3 Evaluation Details and Public Checkpoint Sources

The exact public checkpoints used for the contextual comparison in the main text are listed in

Table S4. Internal evaluations followed the fixed PubMed, Wikipedia, and C4 validation sets described in the main Methods section, whereas public-checkpoint comparisons used the same raw text with each model’s native tokenizer.

**Loss computation.** Perplexity was computed from next-token cross-entropy with padding masked via ignore_index = *−*100. Non-finite losses were excluded from averaging.

Table S4: Exact public checkpoint sources used for the contextual comparison in the main text.

| **Model** | **Public checkpoint** |
| --- | --- |
| BioGPT | microsoft/biogpt |
| BioGPT-Large | microsoft/BioGPT-Large |
| BioGPT-Large-PubMedQA | microsoft/BioGPT-Large-PubMedQA |
| BioMedLM | stanford-crfm/BioMedLM |
| Meditron3-Gemma2-2B | OpenMeditron/Meditron3-Gemma2-2B |
| Bio-Medical-Llama-3.2-1B | ContactDoctor/Bio-Medical-Llama-3-2-1B-CoT-012025 |
| Gemma3-1B (fine-tuned) | kunjcr2/gemma3_finetune |

# S4 Supervised Fine-Tuning Details

Table S5 summarizes the selected supervised fine-tuning hyperparameters for each BioMamba scale. Table S6 provides class-specific yes/no F1 breakdowns for BioASQ and PubMedQA across scales.

Table S5: Supervised fine-tuning hyperparameters. “BioASQ share” denotes the proportion of BioASQ data in the training mixture.

| **Model** | **Learning rate** | **Epochs** | **Effective batch size** | **Weight decay** | **BioASQ share** |
| --- | --- | --- | --- | --- | --- |
| 130M | 3e-5 | 3 | 128 | 0.01 | 0.65 |
| 370M | 3e-5 | 2 | 64 | 0.01 | 0.70 |
| 780M | 2e-5 | 5 | 64 | 0.03 | 0.65 |
| 1.3B | 7e-6 | 3 | 32 | 0.03 | 0.70 |
| 2.7B | 2e-5 | 5 | 32 | 0.01 | 0.65 |

# S5 MIMIC-IV Clinical SFT and Evaluation Details

This section provides full details for the MIMIC-IV clinical generation experiments reported in the main text.

## S5.1 Data source and splits

The evaluation corpus is MIMIC-IV-Note v2.2 [34], derived from the broader MIMIC-IV resource [35], and contains 331,793 de-identified discharge summaries from 145,914 unique patients (average note length: 10,551 characters). Data were split by subject_id at a 9:1 ratio (seed = 42), yielding approximately 131,323 training patients (298,614 notes) and 14,591 test patients (33,179 notes).

## S5.2 SFT data construction

Two supervised fine-tuning tasks were constructed from the discharge notes:

**Note completion.** The first 50% of each note serves as the instruction; the remainder is the response. Filtering requires *≥*100 characters for the prefix and *≥*50 characters for the continuation Table S6: Class-specific yes/no F1 for BioASQ and PubMedQA across model scales. M2+SFT

= Mamba2+SFT (the public Mamba2 base checkpoint after supervised fine-tuning); BM+SFT = BioMamba+SFT (the counterpart after continued pretraining and the same supervised fine-tuning).

Accuracy and macro-F1 are reported in the main text (Table 12); this table shows only the yes/no F1 breakdown to avoid repetition.

| **Benchmark** | **Scale** | **Arm** | **Yes F1** | **No F1** |
| --- | --- | --- | --- | --- |
| BioASQ 13B | 130M | M2+SFT  BM+SFT | 0.513 **0.768** | 0.558  0.500 |
|  | 370M | M2+SFT  BM+SFT | **0.862**  0.850 | 0.585 **0.591** |
|  | 780M | M2+SFT  BM+SFT | **0.847**  0.835 | 0.609 **0.721** |
|  | 1.3B | M2+SFT  BM+SFT | 0.877 **0.893** | 0.720 **0.769** |
|  | 2.7B | M2+SFT  BM+SFT | **0.927**  **0.927** | **0.852**  **0.852** |
| PubMedQA | 130M | M2+SFT  BM+SFT | 0.667 **0.727** | 0.472 **0.595** |
|  | 370M | M2+SFT  BM+SFT | 0.736 **0.746** | **0.639**  0.633 |
|  | 780M | M2+SFT  BM+SFT | 0.737 **0.754** | 0.636 **0.667** |
|  | 1.3B | M2+SFT  BM+SFT | 0.737 **0.782** | 0.619 **0.697** |
|  | 2.7B | M2+SFT  BM+SFT | 0.783 **0.800** | 0.703 **0.745** |

(max_context_chars = 3,000; max_response_chars = 2,000). Up to 20,000 samples were retained.

**Discharge summary generation.** Admission sections (chief complaint, history of present illness, past medical history, medications, allergies, social and family history, physical examination, and laboratory results) form the instruction. Discharge sections (hospital course, assessment and plan, discharge diagnosis, discharge medications, discharge instructions, and discharge condition) form the response. The same character limits and filters apply, yielding approximately 12,893 valid samples.

The combined dataset contains 32,893 samples (90% train / 10% validation). On average each sample contains 1,023 total tokens, of which 139 (13.6%) are supervised response tokens. Instruction masking sets the loss label of instruction tokens to *−*100.

## S5.3 SFT hyperparameters

Table S7 reports the Mamba2+SFT and BioMamba+SFT configurations at 130M, 370M, 780M, and

1.3B after hyperparameter tuning. The 2.7B configurations for both arms were selected by a small sweep over learning rates and epochs following the same conservative pattern; the exact selected recipes and training logs are in the public code release (Supplementary Section S15).

Table S7: MIMIC-IV clinical SFT configuration for both arms at 130M–1.3B. Mamba2+SFT uses the baseline recipe; BioMamba+SFT uses lower learning rates and more epochs to preserve domain knowledge acquired during continued pretraining. All runs use AdamW with weight decay 0.01, cosine learning-rate schedule, bf16 precision, gradient clipping 1.0, and early stopping with patience 5; Mamba2+SFT uses 10% warmup throughout. The 2.7B recipe for both arms is in the public code release (Supplementary Section S15).

### Recipe Size Batch/GPU Grad Effective Learning Epochs Warmup

**Accum	BS	Rate**

| Mamba2+SFT | 130M  370M  780M | 8  4  4 | 4  8  8 | 32  32  32 | 2e-5  2e-5  2e-5 | 3  3  3 | 0.10 0.10  0.10 |
| --- | --- | --- | --- | --- | --- | --- | --- |
|  | 1.3B | 2 | 16 | 32 | 2e-5 | 3 | 0.10 |
| BioMamba+SFT | 130M  370M  780M | 8  16  16 | 4  2  2 | 32  32  32 | 1e-5  1e-5  7.5e-6 | 5  5 6 | 0.10 0.10  0.12 |
|  | 1.3B | 16 | 2 | 32 | 9e-6 | 6 | 0.12 |

## S5.4 Evaluation protocol

Evaluation used 500 randomly sampled test notes per task from the patient-level test split. Both tasks generated up to 128 tokens with greedy decoding (temperature = 0). The maximum context length was 4,000 characters. ROUGE scores were computed using the rouge-score library with stemming enabled.

## S5.5 BioMamba+SFT tuning observations

A key finding during hyperparameter tuning is that validation loss does not reliably predict ROUGE quality for BioMamba+SFT models. Lower learning rates often produce higher validation loss but better ROUGE scores. Different model sizes require different learning rates: smaller models (130M–370M) perform best with 1e-5, whereas the 780M and 1.3B models require rates between 7.5e-6 and 9e-6. This suggests that BioMamba models have already internalized domain knowledge and are more sensitive to learning-rate magnitude during clinical fine-tuning.

# S6 Additional MIMIC-IV Clinical Generation Case Studies

To complement the representative main-text case studies, we present four additional qualitative examples from the 1.3B model comparing three training stages: Mamba2 (pretrained only), Mamba2+SFT (supervised fine-tuning without continued pretraining), and BioMamba+SFT (continued pretraining followed by supervised fine-tuning). All examples use greedy decoding with a maximum of 128 new tokens.

## S6.1 Additional note completion examples

In the note completion task, the model receives the first 50% of a discharge note and generates a continuation.

**Laboratory results continuation.** The input ends with a partial complete blood count. Table S8 shows that the base model degenerates into repetitive entries, Mamba2+SFT produces correct but incomplete results, and BioMamba+SFT generates three distinct lab panels (basic metabolic panel, minerals, and liver function tests).

Table S8: Additional note-completion case: laboratory results continuation. Input ends with “...WBC-6.2 RBC-3.23* Hgb-9.7* Hct-30.6* MCV-95 MCH-30.1”.

| **Source** | **Text** | **R-1** |
| --- | --- | --- |
| Reference | MCHC-31.8 RDW-15.2 Plt ___ *→* Glucose-70 UreaN-40* Creat-2.4* Na-145 K-4.5 Cl-115* HCO3-21* *→* Brief Hospital Course: ___ year old woman with IDDM, HTN, CKD stage IV... | — |
| Mamba2 | MCHC-32.2* RDW-0.9*... then degenerates into repetitive meaningless entries (“BUN-14.1*” “BIL-0.9*” repeated). | 3.96 |
| Mamba2+SFT | MCHC-31.8, generates WBC differential (Neuts/Lymphs/Monos), Glucose-137* UreaN-25* Creat-1.9* — correct format but missing electrolytes. | 5.17 |
| **BioMamba+SFT** | MCHC-31.0, then generates **complete BMP** (Glucose-137* UreaN-24* Creat-1.6* Na-141 K-4.2 Cl-102 HCO3-26), **minerals** (Calcium-8.8 Phos-  3.5 Mg-1.9), and **liver function** (ALT-16 AST-20 AlkPhos-76). | **8.58** |

**Post-operative course continuation.** The input describes post-operative pain management. Table S9 shows that BioMamba+SFT exactly reproduces the standardized preadmission medication statement used in MIMIC notes and generates a well-structured medication list, achieving ROUGE-1 of 16.43 (+25% over Mamba2+SFT).

Table S9: Additional note-completion case: post-operative course continuation. Input ends with “...His pain was well controlled on minimal amounts of oxycodone. He took Colace and senna”.

| **Source** | **Text** | **R-1** | **R-2** |
| --- | --- | --- | --- |
| Reference | to prevent constipation. Incision was intact without redness or drainage. He was ambulating well. He received teaching for all of his transplant medications. Medications on Admission: 1.  Atorvastatin 10mg PO DAILY 2. Ferrous Sulfat... | — | — |
| Mamba2 | “On postop day 4, he was discharged home on metoprolol tartrate, dulcolax suppository, and oxycodone” — repeats the discharge description twice and gives an incomplete medication list. | 11.47 | 0.93 |
| Mamba2+SFT | “He was discharged home on postop day 4 in stable condition.” *→* generates reasonable admission and discharge medication lists with transplant-appropriate drugs (Cellcept, Solu-medrol). | 13.15 | 4.71 |
| **BioMamba+SFT** | “He was discharged home on postop day 3 in stable condition.”  *→* **exactly reproduces**: “Medications on Admission: The Preadmission Medication list is accurate and complete.” *→* generates a structured medication list (Amlodipine, Aspirin, Atorvastatin, Calcitriol, Docusate, Furosemide). | **16.43** | **9.95** |

## S6.2 Additional discharge-summary generation examples

In the discharge summary generation task, the model receives structured admission sections (chief complaint, history of present illness, physical exam, labs, etc.) and generates discharge content.

**Diverticulitis surgery discharge.** Table S10 shows a clear difference in document planning: BioMamba+SFT spontaneously generates a structured Hospital Course narrative, the most clinically central section of a discharge summary, whereas Mamba2+SFT remains closer to laboratory-style continuation.

**Orthopedic discharge instructions.** Table S11 illustrates the repetition problem: Mamba2+SFT falls into a loop repeating “Please have your INR checked” six or more times, whereas BioMamba+SFT generates concise, non-repetitive instructions covering four distinct categories.

## S6.3 Summary of qualitative findings

Table S12 summarizes the capability progression across the three training stages observed in the main-text and supplementary case studies.

Table S10: Additional discharge-summary case: diverticulitis surgery discharge. Input: Service: SURGERY; Chief Complaint: Abdominal pain and failure to thrive related to diverticulitis; Procedure: Low anterior resection, mobilization of splenic flexure...

| **Source** | **Text** | **R-1** | **R-2** |
| --- | --- | --- | --- |
| Reference | DISCHARGE MEDICATIONS: 1. Acetaminophen 325–650mg PO Q4H:PRN pain 2. Aspirin 81mg PO DAILY 3. Depakote 250mg BID 4. Levothyroxine 75mcg PO DAILY 5. OxycoDONE ___ mg PO Q4H:PRN pain... | — | — |
| Mamba2 | Generates repetitive meaningless lab values (“pH-7.4* O2-95% CO2-38% Hct-38.0*” repeated multiple times). | 2.86 | 0.0 |
| Mamba2+SFT | Generates lab results in correct format, but lacks discharge medications and Hospital Course narrative. | 8.14 | 0.91 |
| **BioMamba+SFT** | First generates lab results, then produces **“Patient is a**  **___ year old woman with a history of diverticulitis who presented with abdominal pain and failure to**  **thrive...”**, spontaneously creating a Hospital Course narrative that accurately summarizes the admission reason and surgical procedure. | **21.55** | **4.35** |

Table S11: Additional discharge-summary case: orthopedic discharge instructions. Input: Service: ORTHOPAEDICS; Allergies: Penicillins / Sulfa / Aspirin / Iodine / NSAIDs / Codeine / Gluten /

Shellfish... (extensive allergy list).

| **Source** | **Text** | **R-1** | **R-2** |
| --- | --- | --- | --- |
| Reference | HOSPITAL COURSE: ...tolerating a regular diet and feeling well... afebrile with stable vital signs... pain was adequately controlled on an oral regimen... | — | — |
| Mamba2 | Repeatedly generates a generic template (“Home ___, dressing changes as instructed, wound checks, staple removal at two weeks”). | 6.50 | 0.0 |
| Mamba2+SFT | Generates DVT prophylaxis (coumadin 4 weeks) and INR monitoring, but **falls into repetition** (“Please have your INR checked on ___” repeated 6+ times). | 11.76 | 3.22 |
| **BioMamba+SFT** | “DVT Prophylaxis: Please take coumadin for 2 weeks.” *→*  “Resume your home medications unless otherwise directed.” *→* “Follow up with your PCP.” *→* “Please follow up with your outpatient physical therapy appointment.” — **concise, wellorganized discharge instructions**. | **14.75** | **5.42** |

Table S12: Qualitative capability comparison across training stages based on 1.3B model case studies.

| **Capability** | **Mamba2** | **Mamba2+SFT** | **BioMamba+SFT** |
| --- | --- | --- | --- |
| Clinical terminology accuracy | Frequent misuse (e.g.,  “RRR” to all systems) | Generally correct | Accurate and comprehensive |
| Repetition avoidance | Severe degeneration | Occasional repetition | Minimal repetition |
| Lab result coverage | Disorganized format | Correct format, incomplete coverage | Correct format, multipanel coverage |
| Discharge document structure | Cannot generate | Generates basic framework | Structured with appropriate content |
| Medication appropriateness | Irrelevant medications | Partially appropriate | Clinically standard regimens |
| Hospital Course narra-  tive | Cannot generate | Fragmented | Coherent clinical narrative |

These additional case studies confirm that the quantitative ROUGE improvements reported in the main text reflect meaningful differences in clinical generation quality. BioMamba+SFT generates more comprehensive clinical content (multi-panel lab results, medication reconciliation, Hospital Course narrative), selects medications aligned with standard care patterns, and produces betterstructured discharge documents with less repetitive degeneration. These qualitative advantages originate from domain knowledge acquired during continued pretraining on PubMed literature, which is then effectively leveraged through clinical supervised fine-tuning.

# S7 Cross-Scale Data-Mixture Ablation at 370M

To verify that the selected 80%/10%/10% PubMed/C4/Wikipedia mixture is not specific to the

130M pilot, we additionally compared the paper recipe against a PubMed-only control at the 370M scale under matched optimizer and scheduler settings (Table S13). The PubMed-only control worsens Wikipedia-style held-out perplexity by 69% and C4 perplexity by 15% relative to the 80%/10%/10% recipe, and is also slightly worse on held-out PubMed (9.32 *→* 9.91). This pattern is consistent with the 130M pilot, where the 80%/10%/10% mixture already slightly outperformed PubMed-only on PubMed (8.41 vs. 8.47; Table 13); the gap is larger at 370M, indicating that domain-only continued pretraining incurs a larger forgetting penalty at scale even on in-domain text. This confirms that the mixture is load-bearing at larger scale and not an artifact of pilot selection.

Table S13: 370M cross-scale validation of the 80%/10%/10% continued pretraining mixture against a PubMed-only control. PubMed-only continued pretraining worsens Wikipedia-style held-out perplexity by +69% and C4 perplexity by +15% relative to the 80%/10%/10% recipe, and is also slightly worse on held-out PubMed (9.32 *→* 9.91), consistent with the 130M pilot pattern (Table 13).

| **370M CPT recipe** | **C4 PPL (***↓***)** | **Wikipedia**  **PPL (***↓***)** | **PubMed**  **PPL (***↓***)** |
| --- | --- | --- | --- |
| 80%/10%/10% (paper recipe) | **18.67** | **16.96** | **9.32** |
| PubMed only | 21.45 | 28.72 | 9.91 |
| ∆ (PubMed-only *−* paper) | +2*.*78 (+15%) | +11*.*76 (+69%) | +0*.*59 |

# S8 Out-of-Domain Evaluation: Protocol and Per-Task Grid

The six out-of-domain multiple-choice benchmarks referenced in Table 4 of the main text—LAMBADA, HellaSwag, ARC-Easy, ARC-Challenge, PIQA, and OpenBookQA—were scored by continuation log-likelihood at a fixed evaluation budget, using each benchmark’s standard split (LAMBADA, HellaSwag, OpenBookQA: 5,000, 5,000, 500 test items; ARC-Easy, ARC-Challenge, PIQA: 570, 299, 1,838 items respectively). The same evaluation script was used for Mamba2 and BioMamba at every scale. Table S14 reports the full per-task accuracy grid (5 scales *×* 6 tasks *×* 2 arms, 60 cells total) that underlies the summary in Table 4 of the main text. All per-task ∆ = (BioMamba *−* Mamba2) lie inside *±*3pp at every scale, and no task shows a systematic regression in either direction. The

370M LAMBADA row is noticeably lower than the 130M and 780M rows for *both* arms (Mamba2 18.70%; BioMamba 19.62%); this is a known pipeline artifact at that particular 370M checkpoint and affects the two arms identically, so the BioMamba*−*Mamba2 comparison (+0*.*92pp) is unaffected. The evaluation script is released with the training and evaluation code (Supplementary Section S15).

Table S14: Full per-task OOD accuracy grid (%) for Mamba2 base and BioMamba across five scales and six benchmarks. Mean is the unweighted average across the six tasks. Boldface marks the arm with the higher accuracy within each (scale, task) cell; a tie is not bolded.

| **Scale** | **Arm** | **LAMBADAHellaSwag** | | **ARC-E** | **ARC-C** | **PIQA** | **OBQA** | **Mean** |
| --- | --- | --- | --- | --- | --- | --- | --- | --- |
| 130M | Mamba2-130M  BioMamba-130M | **53.18**  51.08 | **32.00**  31.68 | **42.63**  41.93 | 27.42 **28.09** | 64.53 **64.85** | 25.00 **25.80** | **40.79**  40.57 |
| 370M | Mamba2-370M  BioMamba-370M | 18.70 **19.62** | 38.88 **39.16** | **46.67**  46.14 | 27.09 **28.76** | 69.21 **69.80** | **27.80**  26.20 | 38.06 **38.28** |
| 780M | Mamba2-780M  BioMamba-780M | **55.52**  54.52 | 45.80 **46.00** | 52.98 **53.51** | **31.10**  30.10 | **72.52**  71.93 | **31.60**  30.40 | **48.26**  47.74 |
| 1.3B | Mamba2-1.3B  BioMamba-1.3B | 51.22 **53.58** | 49.34 **49.64** | 59.65 **60.35** | **35.12**  34.78 | 74.32 **74.76** | 30.60  30.60 | 50.04 **50.62** |
| 2.7B | Mamba2-2.7B  BioMamba-2.7B | **63.86**  63.82 | 52.82 **53.28** | 62.98 **63.16** | **35.45**  34.78 | 76.01 **76.06** | 34.20 **34.80** | 54.22 **54.32** |

# S9 Matched-Budget SFT Control at 1.3B

To verify that the BioMamba+SFT advantage is not an artifact of the more conservative BioMamba SFT schedule, we additionally fine-tuned Mamba2-1.3B with the exact BioMamba SFT recipe reported in Table S7 (lower learning rate, more epochs, same effective batch size). Table S15 shows that the BioMamba schedule does not transfer the BioMamba gain to Mamba2; instead, it hurts Mamba2 relative to its own recipe on both completion and discharge generation. This is the expected behavior if BioMamba+SFT is leveraging information acquired during continued pretraining rather than exploiting an unequal optimization effort.

Table S15: Matched-budget 1.3B MIMIC-IV control: Mamba2-1.3B trained with the exact BioMamba SFT recipe (Table S7). The BioMamba schedule worsens Mamba2 relative to its own recipe, which rules out unequal SFT budgets as a source of the BioMamba+SFT advantage. ROUGE %, 500 test samples, greedy decoding.

| **1.3B arm (recipe)** | **Completion R-**  **1 / R-2 / R-L** | **Discharge R-**  **1 / R-2 / R-L** |
| --- | --- | --- |
| Paper Mamba2+SFT | 7.93 / 3.28 / 5.76 | 9.99 / 3.70 / 7.04 |
| Paper BioMamba+SFT | **8.11** / **3.33** / **5.89** | **10.11** / 3.69 / **7.04** |
| Mamba2 @ BioMamba recipe | 7.56 / 2.77 / 5.28 | 9.00 / 2.58 / 6.14 |
| ∆ vs. paper BioMamba+SFT | *−*0*.*55*/ −* 0*.*56*/ −* 0*.*61 | *−*1*.*11*/ −* 1*.*11*/ −* 0*.*90 |
| ∆ vs. paper Mamba2+SFT | *−*0*.*37*/ −* 0*.*51*/ −* 0*.*48 | *−*0*.*99*/ −* 1*.*12*/ −* 0*.*90 |

# S10 Three-Seed Reproduction on BioASQ and PubMedQA

To assess the sensitivity of BioMamba+SFT QA accuracy to random seed and to interpret the single-question BioASQ deltas at 370M and 2.7B, we reproduced BioMamba+SFT at every scale across seeds *{*13*,*42*,*97*}* (Table S16). Reproduced means stay within seed noise of the paper values on BioASQ (*σ ≤* 0*.*021) and match or exceed the paper on PubMedQA (*σ ≤* 0*.*032). Because the BioASQ test split contains only 82 items, a single-question difference corresponds to 1.22 percentage points, which matches the magnitude of the 370M and 2.7B deltas reported in Table 12.

Table S16: Three-seed reproduction of BioMamba+SFT accuracy on BioASQ and PubMedQA across all five scales (seeds *{*13*,*42*,*97*}*). Paper columns report the single-seed values reported in the main text. ∆ columns report the difference between the three-seed mean and the paper value. BioASQ *n* = 82; PubMedQA *n* = 200.

| **Scale** | **3-seed**  **mean** *±* **std** | **BioASQ**  **Paper** | ∆ **(pp)** | **PubMedQA** | |  |
| --- | --- | --- | --- | --- | --- | --- |
|  |  |  |  | **3-seed**  **mean** *±* **std** | **Paper** | ∆ **(pp)** |
| 130M | 0*.*683 *±* 0*.*000 | 0.683 | +0*.*00 | 0*.*630 *±* 0*.*003 | 0.630 | +0*.*00 |
| 370M | 0*.*780 *±* 0*.*012 | 0.780 | +0*.*00 | 0*.*685 *±* 0*.*013 | 0.660 | +2*.*50 |
| 780M | 0*.*817 *±* 0*.*021 | 0.793 | +2*.*44 | 0*.*678 *±* 0*.*023 | 0.675 | +0*.*30 |
| 1.3B | 0*.*841 *±* 0*.*012 | 0.854 | *−*1*.*22 | 0*.*713 *±* 0*.*032 | 0.700 | +1*.*30 |
| 2.7B | 0*.*898 *±* 0*.*007 | 0.902 | *−*0*.*40 | 0*.*730 *±* 0*.*012 | 0.730 | +0*.*00 |

# S11 Item-Level Error Analysis on BioASQ

To complement the variance analysis, we ran a per-example wrong-index analysis comparing Mamba2+SFT and BioMamba+SFT on the BioASQ test split at every scale (Table S18). At every scale, BioMamba fixes at least as many items as it breaks: the fixes/breaks ratios are 9:5 at 130M, 6:5 at 370M, 2:0 at 780M, 4:1 at 1.3B, and 5:3 at 2.7B. Most errors that remain are shared between the two arms—at 780M, 100% of BioMamba’s errors are also made by Mamba2, and at 1.3B the share is 11 of 12—so residual disagreements at larger scales are confined to a handful of genuinely hard items rather than indicating a regression attributable to continued pretraining.

Table S17: Raw correct/total counts and 95% Wilson binomial confidence intervals for the BioMamba+SFT accuracy values reported in Table 12. BioASQ test-set size *n* = 82; PubMedQA test-set size *n* = 200. Confidence-interval endpoints are in percentage points.

## BioASQ PubMedQA

| **Scale** | **Correct / total (acc.)** | **95% Wilson CI** | **Correct / total (acc.)** | **95% Wilson CI** |
| --- | --- | --- | --- | --- |
| 130M | 56/82 (68.29%) | [57*.*6*,*77*.*4] | 126/200 (63.00%) | [56*.*1*,*69*.*4] |
| 370M | 64/82 (78.05%) | [67*.*9*,*85*.*6] | 132/200 (66.00%) | [59*.*2*,*72*.*2] |
| 780M | 65/82 (79.27%) | [69*.*3*,*86*.*6] | 135/200 (67.50%) | [60*.*7*,*73*.*6] |
| 1.3B | 70/82 (85.37%) | [76*.*1*,*91*.*4] | 140/200 (70.00%) | [63*.*3*,*75*.*9] |
| 2.7B | 74/82 (90.24%) | [81*.*9*,*95*.*0] | 146/200 (73.00%) | [66*.*5*,*78*.*7] |

Table S18: BioASQ item-level wrong-index analysis across five scales, computed on the three-seed reproduction runs (Supplementary Table S16) rather than the single-seed values in Table 12. “Shared wrong” counts items that both arms get wrong; “BM fixes” counts items Mamba2+SFT gets wrong but BioMamba+SFT gets correct; “BM breaks” counts items Mamba2+SFT gets correct but BioMamba+SFT gets wrong; fixes minus breaks therefore corresponds to the three-seed accuracy delta (in items on the 82-item test split), not to the paper’s single-seed ∆.

| **Scale** | **Shared wrong** | **BM fixes** | **BM breaks** | **BM errors shared with M2** |
| --- | --- | --- | --- | --- |
| 130M | 22 | 9 | 5 | 22 / 27 (81%) |
| 370M | 15 | 6 | 5 | 15 / 20 (75%) |
| 780M | 17 | 2 | 0 | 17 / 17 (100%) |
| 1.3B | 11 | 4 | 1 | 11 / 12 (92%) |
| 2.7B | 5 | 5 | 3 | 5 / 8 (63%) |

# S12 NLI Factuality Protocol and Auto-Flagged Candidate List

The NLI-ensemble factuality proxy reported in the main text (Table 10) uses two MNLI checkpoints

(roberta-large-mnli and microsoft/deberta-v2-xlarge-mnli), applied across 500 2.7B MIMIC discharge generations per arm and per decoding setting. For each (generation, source) pair we scored entailment, neutral, and contradiction probabilities under each MNLI checkpoint. A generation is flagged as contradictory only when *both* checkpoints independently assign contradiction probability *>* 0*.*7 (unanimous-agreement rule, equivalent to an AND over the two scorers); this is the tiebreak-free specialization of plain majority voting for a two-model ensemble and is conservative, since contradiction is the minority class. Alternative rules—e.g. averaging the two contradiction probabilities and thresholding at 0*.*7, or OR-agreement with any one checkpoint flagging contradiction— yield higher baseline contradiction rates but do not change the relative ordering of the two arms at the decoding settings tested. The source passed to the NLI scorers is the input admission text only—chief complaint, history of present illness, past medical history, medications, allergies, social and family history, physical examination, and laboratory/imaging summaries—i.e. the same sections used as the instruction for discharge summary generation. Discharge-section text (hospital course, discharge diagnosis, discharge medications, discharge instructions) is never passed to the scorer as source, so this is an input-grounding check rather than a comparison against the reference discharge. The hypothesis is the model’s 128-token continuation.

We additionally exported a clinician-review candidate list, in which auto-flagged sentences are organized into four categories—factual (verifiable clinical content contradicted by the source), omission (important source content missing from the generation), fabrication (plausible-looking content with no source support), and safety (medication- or dosage-related generations). This list is a proxy for factuality review rather than a clinician audit, and the auto-flagging pipeline is intended as a precursor to clinician involvement rather than a replacement for it. A copy of the list is available from the authors upon request.

# S13 MIMIC-IV Discharge Section-Header F1 (1.3B and 2.7B)

The lightweight section-header F1 diagnostic (Table S19) is a structural rather than factual proxy, measuring how often generations contain the discharge section headers (Hospital Course, Discharge Medications, Discharge Instructions, etc.) that a reference discharge note contains.

We report this at 1.3B and 2.7B under greedy and both nucleus settings; BioMamba leads or ties Mamba2 across all six (scale *×* decoding) cells tested. The main-text diagnostic table (Table 9) already covers distinct-*n* and repeated-4-gram rate at 1.3B; greedy is omitted there because under argmax both arms collapse to similar low-diversity outputs, making these metrics uninformative between arms.

Table S19: Discharge section-header F1 (a lightweight structure diagnostic, not a factuality metric). BioMamba leads or ties Mamba2 across all six tested (scale *×* decoding) cells.

| **Scale** | **Decoding** | **M2+SFT F1** | **BM+SFT F1** | ∆ **(BM** *−* **M2)** |
| --- | --- | --- | --- | --- |
| 1.3B | greedy | 0.285 | **0.293** | +0*.*008 |
| 1.3B | nucleus-0.9 | 0.259 | **0.260** | +0*.*001 |
| 1.3B | nucleus-0.95 | 0.190 | **0.235** | +0*.*045 |
| 2.7B | greedy | 0.168 | **0.196** | +0*.*028 |
| 2.7B | nucleus-0.9 | 0.170 | **0.188** | +0*.*019 |
| 2.7B | nucleus-0.95 | 0.152 | **0.158** | +0*.*006 |

# S14 Bootstrap Confidence Intervals and Paired Bootstrap at 2.7B

For the 2.7B MIMIC-IV row in Table 6, we report two complementary bootstrap analyses with 1000 resamples each over the 500 test samples. Table S20 shows 95% percentile-bootstrap confidence intervals for per-arm ROUGE-1 and ROUGE-L; Table S21 shows paired-bootstrap significance on per-sample ROUGE-1 *differences* between BioMamba+SFT and Mamba2+SFT. On both tasks, BioMamba-2.7B+SFT lies above Mamba2-2.7B+SFT on ROUGE-1 and ROUGE-L (Table S20), and the paired bootstrap gives *P*(∆ *>* 0) = 1*.*00 for completion and *P*(∆ *>* 0) = 0*.*997 for discharge (Table S21), indicating that the 2.7B advantage is not within test-sample noise. The per-arm absolute CIs in Table S20 overlap between the two arms; the paired-bootstrap analysis in Table S21 is therefore the more informative significance test because it exploits within-sample pairing.

Table S20: Per-arm 95% percentile-bootstrap confidence intervals for ROUGE-1 and ROUGE-L at 2.7B on MIMIC-IV clinical generation (500 test samples, 1000 resamples; greedy decoding).

| **2.7B arm** | **Task** | **R-1** | **R-1 95% CI** | **R-L** | **R-L 95% CI** |
| --- | --- | --- | --- | --- | --- |
| Mamba2+SFT | completion | 7.16 | [6*.*72*,*7*.*63] | 4.98 | [4*.*72*,*5*.*30] |
| **BioMamba+SFT** | completion | **7.57** | [7*.*16*,*8*.*02] | **5.30** | [4*.*97*,*5*.*62] |
| Mamba2+SFT | discharge | 8.87 | [8*.*17*,*9*.*63] | 6.21 | [5*.*66*,*6*.*76] |
| **BioMamba+SFT** | discharge | **9.39** | [8*.*69*,*10*.*09] | **6.46** | [5*.*95*,*7*.*07] |

Table S21: Paired-bootstrap significance of the 2.7B BioMamba+SFT advantage over Mamba2+SFT on per-sample ROUGE-1 differences (500 test samples, 1000 resamples; greedy decoding).

| **Task** | **Mean** ∆ **R-1** | **95% CI** | *P*(∆ *>* 0) |
| --- | --- | --- | --- |
| Note completion | +0*.*41 | [+0*.*22*,*+0*.*60] | **1.00** |
| Discharge generation | +0*.*52 | [+0*.*14*,*+0*.*88] | **0.997** |

# S15 Reproducibility Details

This section provides additional reproducibility information.

**Base-model identifiers.** The five base Mamba2 checkpoints used for continued pretraining are state-spaces/mamba2-130m, state-spaces/mamba2-370m, state-spaces/mamba2-780m, state-spaces/mamba2-1.3b, and state-spaces/mamba2-2.7b. All internal comparisons reuse the shared GPT-NeoX tokenizer from state-spaces/mamba-2.8b-hf (vocabulary size 50,280).

**Corpus snapshots.** The continued pretraining corpus was loaded from Hugging Face with fixed repository, config, and revision identifiers: PubMed/MEDLINE from cyrilzakka/pubmed-medline (default, revision 432681e19469e93e6c42878d5f41fec400974fb8; the dataset card records date accessed as 2025-05-02), Wikipedia from wikimedia/wikipedia (20231101.en, revision b04c8d1ceb2f5cd4588862100d08de323dccfbaa), and C4 from allenai/c4 (en, revision 1588ec454efa1a09f29cd18ddd04fe05fc8653a2). All three sources were tokenized with the shared tokenizer and filtered to remove near-duplicates and sequences with unreadable characters before truncation/padding to 1,024 tokens. For the fixed held-out perplexity sets, we sampled 1,000 sequences per source with seed 42 from the same PubMed and C4 pools used for continued pretraining but excluded them from training; for the Wikipedia-style held-out evaluation we used the Wikitext-103 raw split (wikitext-103-raw-v1), as described in Section 2.

**MIMIC-IV preprocessing and splits.** MIMIC-IV-Note v2.2 was split by subject_id (9:1 ratio, seed = 42), yielding approximately 131,323 training patients and 14,591 test patients; quantitative evaluation used 500 randomly sampled held-out notes per task. Preprocessing filters (minimum lengths, maximum characters) are reported in Supplementary Section S5.

**Model-selection criteria.** For continued pretraining, the checkpoint with the lowest validation loss was kept for downstream evaluation. For BioMamba+SFT and Mamba2+SFT, the checkpoint with the best downstream ROUGE on the 10% validation split was selected, because validation loss was not a reliable proxy for ROUGE in the BioMamba setting (Section 2; Supplementary Section S4). For QA fine-tuning, the checkpoint with the best validation accuracy was kept.

**Prompt templates.**	Figure S1 shows the exact prompt templates used for yes/no QA fine-tuning, note completion, and discharge summary generation.

**Public release.** The five BioMamba checkpoints (130M / 370M / 780M / 1.3B / 2.7B) and the full training and evaluation code are publicly available at [https://github.com/LeoYML/BioMamba;](https://github.com/LeoYML/BioMamba) the released checkpoints are also collected at [https://huggingface.co/collections/zmzfpc/biomamba.](https://huggingface.co/collections/zmzfpc/biomamba) The release is under the Apache-2.0 license, matching the upstream Mamba2 checkpoints. The repository includes training configurations, preprocessing scripts, and evaluation scripts.

**Yes/No QA (BioASQ / PubMedQA).**

Question: <question>

Context: <context>

Answer (yes or no):

**Note completion (MIMIC-IV).**

Complete the following discharge note.

Beginning of note:

<first 50% of the discharge note>

Continuation:

**Discharge summary generation (MIMIC-IV).**

Generate the discharge sections for the following admission.

Admission sections:

<chief complaint, HPI, PMH, medications, allergies, social/family history, physical exam, labs>

Discharge sections:

Figure S1: Prompt templates used for BioASQ/PubMedQA yes/no question-answering fine-tuning, MIMIC-IV note completion, and MIMIC-IV discharge summary generation. Instruction masking was applied during training so that only response tokens contributed to the loss.
